# Development of an antigen-based approach to noninvasively image CAR T cells in real time and as a predictive tool

**DOI:** 10.1126/sciadv.adn3816

**Published:** 2024-09-18

**Authors:** Julia Fröse, Jennifer Rowley, Ali Salehi Farid, Taha Rakhshandehroo, Paul Leclerc, Howard Mak, Harris Allen, Heydar Moravej, Leila Munaretto, Luis Millan-Barea, Elisabeth Codet, Hannah Glockner, Caron Jacobson, Michael Hemann, Mohammad Rashidian

**Affiliations:** ^1^ David H. Koch Institute for Integrative Cancer Research, Cambridge, MA 02142, USA.; ^2^ Department of Biology, Massachusetts Institute of Technology, Cambridge, MA 02142, USA.; ^3^ Department of Cancer Immunology and Virology, Dana-Farber Cancer Institute, Boston, MA 02215, USA.; ^4^ Harvard Medical School, Boston, MA 02215, USA.; ^5^ Department of Radiology, Brigham and Women’s Hospital, Harvard Medical School, Boston, MA 02215, USA.; ^6^ Parker Institute for Cancer Immunotherapy, San Francisco, CA 94129, USA.

## Abstract

CAR T cell therapy has revolutionized the treatment of a spectrum of blood-related malignancies. However, treatment responses vary among cancer types and patients. Accurate monitoring of CAR T cell dynamics is crucial for understanding and evaluating treatment efficacy. Positron emission tomography (PET) offers a comprehensive view of CAR T cell homing, especially in critical organs such as lymphoid structures and bone marrow. This information will help assess treatment response and predict relapse risk. Current PET imaging methods for CAR T require genetic modifications, limiting clinical use. To overcome this, we developed an antigen-based imaging approach enabling whole-body CAR T cell imaging. The probe detects CAR T cells in vivo without affecting their function. In an immunocompetent B cell leukemia model, CAR-PET signal in the spleen predicted early mortality risk. The antigen-based CAR-PET approach allows assessment of CAR T therapy responses without altering established clinical protocols. It seamlessly integrates with FDA-approved and future CAR T cell generations, facilitating broader clinical application.

## Teaser

Antigen-based CAR-PET imaging detects CD19 CAR T cells with clarity, offering insights into the efficacy of CAR-T therapy.

## INTRODUCTION

Chimeric antigen receptor (CAR) T cell therapy has shown remarkable promise in the treatment of several hematologic malignancies (*1*–*4*). CARs consist of an extracellular antibody fragment designed to recognize tumor-associated or -specific antigens coupled with intracellular signaling motifs to facilitate T cell activation. Notably, CAR T cells can recognize surface structures on cells independently of a specific major histocompatibility complex molecule, making them suitable for patients with tumors expressing the CAR’s target antigen. CAR T cell therapies cover a wide array of antigens, some of which have demonstrated substantial potential (*1*, *3*). To date, six CAR T cell therapies have received Food and Drug Administration (FDA) approval, with four targeting CD19 and two targeting B cell maturation antigen (BCMA) (*5*).

CD19, a type I transmembrane glycoprotein, is pivotal for B lymphocyte development and survival and is widely expressed across human B lineage cells, except for plasma cells (*6*). It serves as both a valuable biomarker and a key therapeutic target for B cell leukemia and lymphoma (*7*, *8*). CAR T cells designed to target CD19 have demonstrated remarkable safety and efficacy in treating acute lymphoblastic leukemia (ALL), leading to complete remission with the absence of minimal residual disease in 67 to 85% of patients given CD19 CAR therapy (*9*–*15*). However, long-term observations reveal a major concern (*10*): While CD19 CAR T cell therapy was highly effective for a substantial proportion of patients with various B cell non-Hodgkin lymphomas, such as large B cell lymphoma, mantle cell lymphoma, and follicular lymphoma, relapse occurred in 40 to 50% of patients (*1*, *10*, *12*, *16*). CD19-negative relapses are often attributed to immune evasion or antigen loss (*17*–*19*), whereas CD19-positive relapses, which constitute more than 50% of relapses, primarily result from the lack of CAR T cell persistence or their diminished antitumor activity (*1*, *10*, *12*, *16*).

Noninvasive methods that monitor CAR T cell dynamics and persistence in vivo are of critical importance to both assessing individual patients’ responses to CAR therapy in real time and to guiding improvements of CAR T cell therapies more broadly. Commonly used methods to assess CAR T cell therapy effectiveness and persistence include detection of cytokines (*20*), digital polymerase chain reaction analysis (*21*), or flow cytometry on blood samples (*22*). However, these methods do not provide information on the biodistribution of infused CAR T cells, and the blood analyses may not accurately quantify the extent of tumor infiltration and expansion. Therefore, these methods do not always correlate with CAR T cell response or persistence (*23*). Thus, more reliable and predictive CAR T cell detection and monitoring techniques are needed.

Whole-body imaging techniques such as positron emission tomography (PET) can be used to tackle this issue. Several PET imaging probes have already been used to assess various aspects of cancer biology in patients, including predicting and assessing treatment response (*24*–*27*). The implementation of CAR T cell imaging can yield crucial insights into treatment responses, providing valuable data for predicting long-term outcomes and enhancing our understanding of associated toxicities, such as cytopenias.

Two major noninvasive imaging methods for CAR T cells have been developed: (i) ex vivo radiolabeling of cells, in which CAR T cells are labeled with a PET radioisotope before adoptive transfer (*28*–*30*) and (ii) in vivo labeling, in which CAR T cells are labeled subsequent to infusion using a small-molecule PET tracer specific for a reporter gene that is coexpressed with the CAR construct (*31*–*36*). Ex vivo labeling provides a simple established method of labeling but requires rapid imaging (up to a few days after injection) due to dilution and effluxion of the labeling agent, a major constraint to clinical applicability. In vivo labeling, however, allows imaging at any time point with longitudinal tracking of CAR T cells and, therefore, more clinically translatable. Several PET reporter systems have been developed (*31*, *33*, *37*–*50*). A PET reporter system was developed using CD19 CAR T cells coexpressing prostate-specific membrane antigen (PSMA) that could be detected using [^18^F]-DCFPyL as a targeted PET tracer (*51*, *52*). CAR T cells were tracked in vivo to the tumor site without any effect on CAR T cell function. The authors found no correlation between CAR T cell presence in peripheral blood versus PET signal at the tumor site, indicating that the peripheral blood may not reflect the degree to which CAR T cells infiltrate into the tumor and that a more clinically relevant assessment can be determined by PET. They found that PET signal accurately detected tumor infiltration of CAR T cells and correlated to response (*51*). Furthermore, the in vivo labeling platform has been tested in the clinic with glioblastoma multiforme patients receiving interleukin-13Rα2 (IL-13Rα2) CAR T cells coexpressing herpes simplex virus–thymidine kinase (HSV-TK), which was detected using [^18^F]-FHBG as a targeted PET tracer. They found that the imaging tracer was safe and effective at tracking CAR T cell infiltration (*45*, *53*).

Notable advancements have been achieved in reporter gene technology (*54*, *55*). Nevertheless, this technology faces notable limitations, including potential immunogenicity [e.g., HSV-TK, *Escherichia coli* dihydrofolate reductase (*48*), and DOTA-based antibody reporters (*56*, *57*)]. Moreover, physiologic expression in several normal organs can lead to background signal [e.g., PSMA (*51*), somatostatin receptor-2 (*58*), and human sodium-iodine symporter (*49*, *50*)]. Common drawbacks further encompass the complexities in design and operation, promoter silencing, and the potential for DNA modification–induced mutations (*59*, *60*). Therefore, despite extensive efforts, the clinical translation of reporter gene–based PET approaches has been limited. Activation marker–based imaging methods, such as Inducible T-cell costimulator (ICOS)–based imaging (*61*), can provide valuable information on the biodistribution of CAR T cells during the response phase. However, these techniques are limited in specificity and sensitivity because markers like ICOS are heterogeneously expressed and not exclusive to CAR T cells. In addition, these methods may not effectively image CAR T cells in their resting state after the initial response, which is crucial for assessing CAR T cell persistence.

To address these limitations, we developed an antigen-based approach using the CAR’s target antigen as the PET probe. This approach offers several advantages similar to the reporter gene method, such as the ability to perform imaging at any time point, rapid radiotracer clearance, and high sensitivity and specificity. It overcomes the limitations associated with the reporter gene method as it does not require any modification of the CAR T cells themselves. Notably, the method can be seamlessly applied to currently FDA-approved CAR T cells without necessitating any changes to the established clinical protocols for CAR T cell therapy. Our findings demonstrate the probe’s ability to detect CD19 CAR T cells in the spleen and bone marrow, serving as a predictive indicator for the CAR T therapy response.

## RESULTS

### Design and synthesis of the antigen-based probe for noninvasive imaging of CD19 CAR T cells

**Fig. 1.**
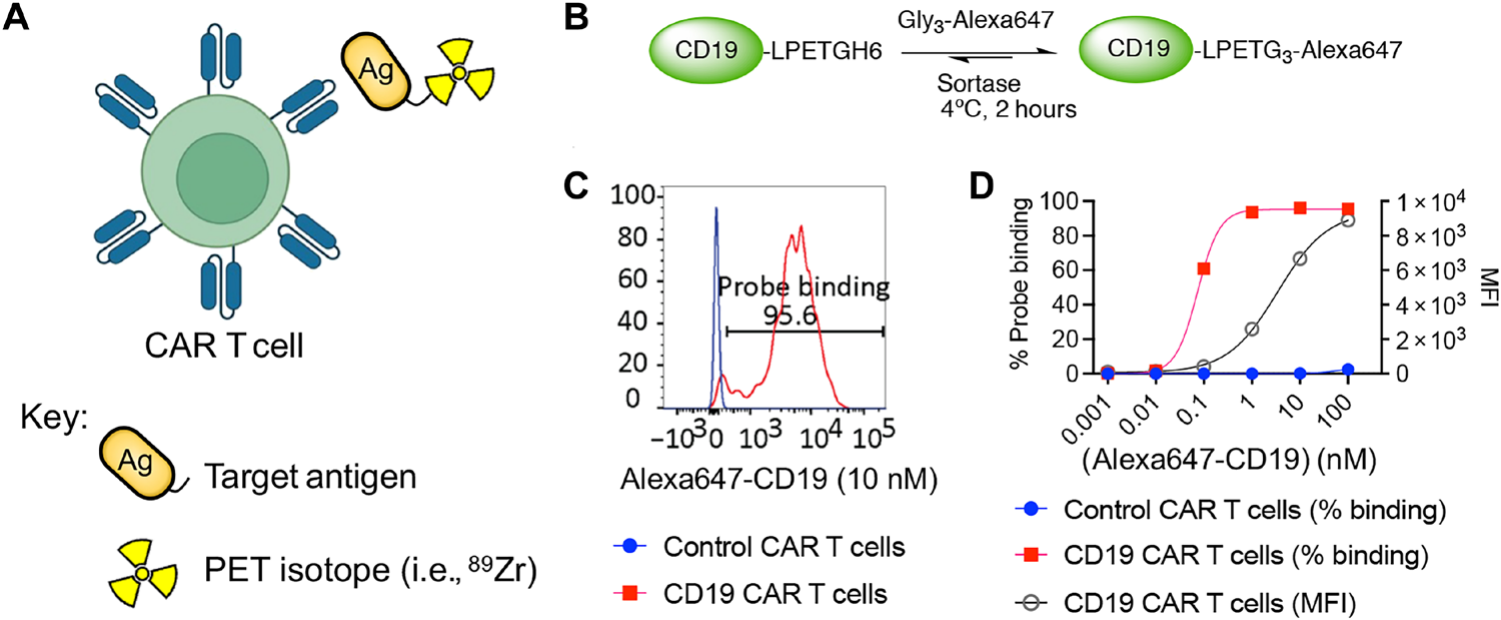
Development of an antigen-based CD19 PET probe for CAR T cell imaging. (**A**) Schematic of the antigen-based strategy for imaging CAR T cells. (**B**) Site-specific labeling of the human CD19 ectodomain, referred to as the CD19 probe here, with Alexa647 fluorophore at the C terminus via a sortase reaction. (**C**) Flow cytometric analysis histograms displaying specific staining of CD19 CAR T cells, but not control CAR T cells, when stained with the Alexa647-labeled CD19 probe at 10 nM. (**D**) Dose-dependent analysis of Alexa647-labeled CD19 probe binding to CD19 CAR T cells (EC_50_ = 0.074 nM) and control (EGFRvIII) CAR T cells (left *y* axis) and dose-dependent mean fluorescent intensity (MFI) of the probe binding to CD19 CAR T cells (right *y* axis) (*n* = 3 for each data point; the experiment was repeated twice with similar results). A curve was fitted for each binding curve, and the EC_50_ was calculated using GraphPad Prism. Error bars represent SD. The error bars are minimal and therefore not visible on our graph. The CAR T cells used in this study are murine T cells transduced with a CAR construct containing an anti-human CD19 ectodomain and a murine CD28-CD3z endodomain.

CD19 is a biomarker of both normal and neoplastic human B lineage cells and is an important target for CAR T cell therapies in B cell leukemias and lymphomas (*6*). To facilitate the precise targeting and monitoring of CD19 CAR T cells in vivo, we postulated that the ectodomain of CD19 (~32 kDa), the portion of the CD19 protein (~58 kDa) that the CD19 CAR targets, could serve as an ideal PET imaging probe (Fig. 1A). Subsequently, we produced the soluble human CD19 ectodomain (*62*) (referred to as “CD19 probe” hereafter) using a human embryonic kidney (HEK) 293T mammalian expression system. The CD19 probe was engineered with a C-terminal FLAG tag (“DYKDDDDK” sequence) to facilitate its characterization using secondary antibodies, a His6-tag for purification, and a sortase recognition motif (“LPETG”) for site-specific modifications (Fig. 1B). The sortase enzyme, a bacterial transpeptidase, recognizes the “LPETG” motif, catalyzing the cleavage of the T─G bond to form an acyl intermediate. Upon addition of a “Gly_3_-R” substrate, this intermediate evolves into the “protein-LPET-Gly_3_-R” product, wherein R can represent various biomolecules of interest (Fig. 1B) (*63*, *64*). The sortase-based method offers site specificity, stoichiometry, robustness, reproducibility, and high yield. Its site-specific nature ensures minimal disruption to the probe’s structural integrity. Purification of the protein was accomplished through Immobilized Metal Affinity Chromatography (IMAC) column chromatography followed by size exclusion chromatography, resulting in a yield of approximately 10 mg/liter of cultured cells. The protein was further characterized via SDS–polyacrylamide gel electrophoresis (SDS-PAGE) analysis (fig. S1A).

### The CD19 probe binds to CD19 CAR T cells with high affinity and specificity

After generating the CD19 probe, binding to CAR T cells was evaluated using flow cytometry. Murine T cells expressing a CAR targeting human CD19 and a control CAR targeting epidermal growth factor receptor variant III (EGFRvIII), typically found in glioblastoma multiforme (*65*), were used in our experiment. CAR T cells were incubated for 30 min with 10 nM of CD19 probe site-specifically labeled with Alexa647 (Alexa647-CD19) through a sortase reaction (Fig. 1B). This process resulted in strong staining of CD19 CAR T cells, as demonstrated in Fig. 1C. Conversely, the control CAR T cells remained unstained (Fig. 1C and fig. S1B). To determine the half-maximal concentration (EC_50_) required for labeling CD19 CAR T cells, 100,000 CAR T cells targeting CD19 or EGFRvIII were incubated for 30 min with decreasing amounts of Alexa647-labeled CD19 probe, ranging from 100 to 0 nM in 10-fold dilutions (Fig. 1D). The results demonstrated dose-dependent probe binding to CD19 CAR T cells at concentrations exceeding 10 pM, with an EC_50_ falling in the picomolar range (EC_50_ = 0.07 nM).

**Fig. 2.**
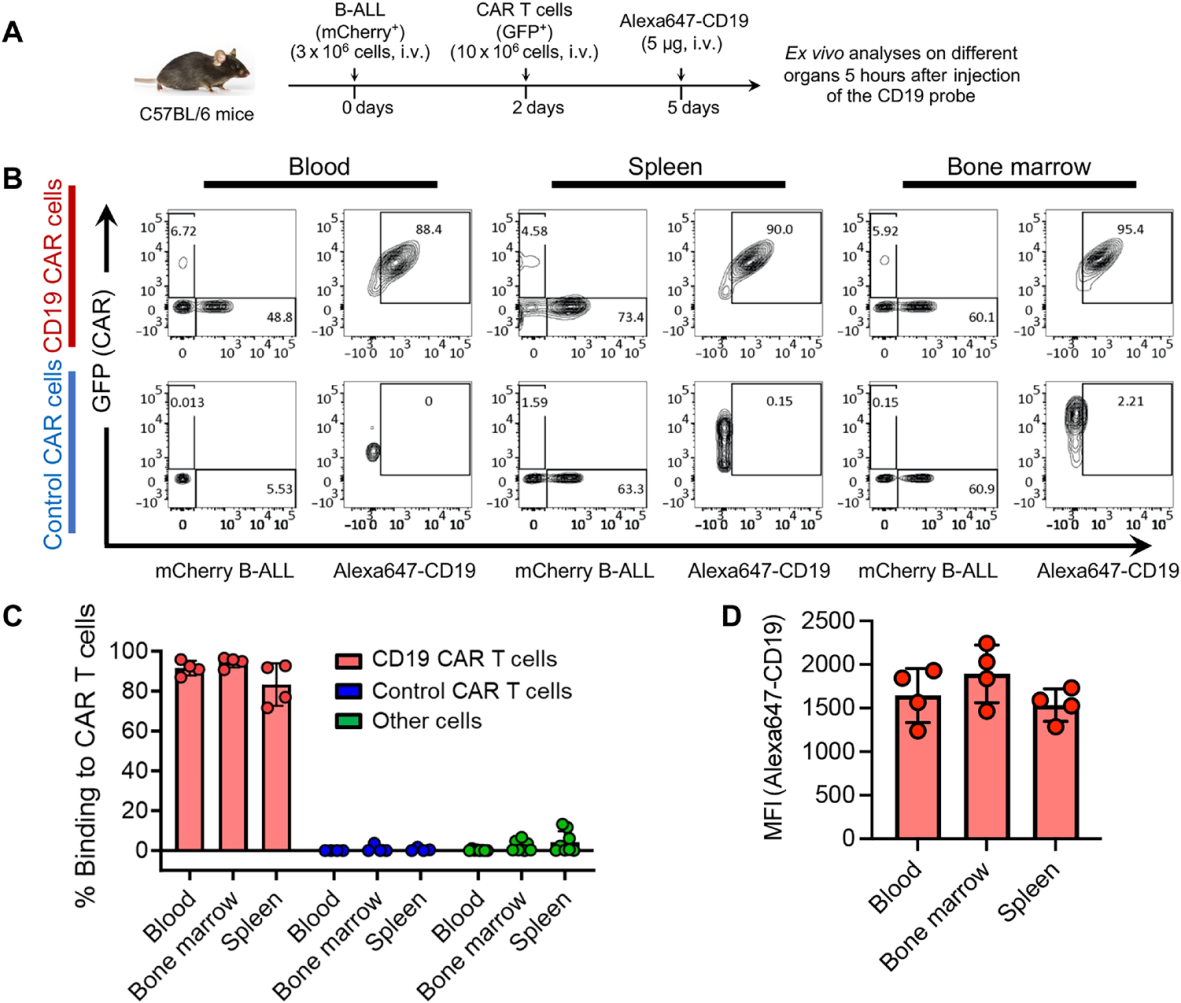
The CD19 probe detects CD19 CAR T cells in vivo with high efficacy and specificity. (**A**) Experimental schematic: C57BL/6 mice received an intravenous (i.v.) injection of 3 million B-ALL cancer cells, followed by a subsequent intravenous injection of freshly prepared CD19 CAR T cells (10 million, expressing anti-human CD19 with a murine CD28-CD3z endodomain CAR construct) or control irrelevant CAR T cells two days later. Five days after the initial injection, the mice were administered the Alexa647-CD19 probe (5 μg, i.v.). Five hours after probe administration, the animals were euthanized, and their organs were collected for flow cytometric analyses. CAR T cells are identified by GFP expression, while B-ALL cells express mCherry. (**B**) Representative flow cytometric analysis of B-ALL cells and CAR T cells in blood, bone marrow, and spleen. CAR T cells stained with the probe are shown in the boxes. Population numbers shown are in %. (**C**) Summary of the efficiency of CD19 probe binding to CD19 CAR T cells compared to control CAR T cells and other cells present in the analyzed organs. (**D**) MFI of CD19 probe binding to CD19 CAR T cells. The data presented are the result of two independent experiments with *n* = 4; error bars indicate SD.

To further investigate these findings, we next assessed the CD19 probe’s ability to bind to CD19 CAR T cells in vivo. We used a transplantable, immunocompetent mouse model of BCR-ABL^+^ B cell ALL (B-ALL), which serves as a preclinical mouse model for the most common type of leukemia treated with CAR T cell therapy (*66*). These B-ALL cells were genetically engineered to display the ectodomain of human CD19 on their cell surface (hCD19^+^ B-ALL). Mice transplanted with these B-ALL cells develop an aggressive form of leukemia and carry a leukemic burden in their blood, bone marrow, and spleen (*67*). In our experiment, we transplanted C57BL/6 mice that had previously received sublethal irradiation [1 × 5 gray (Gy)] with hCD19^+^ B-ALL cells. Two days later, we injected anti-human CD19 CAR T cells or control CAR T cells (anti-EGFRvIII or anti-human CD20) into the tail vein of these mice. Three days after injection of CAR T cells, we administered a tail vein injection of 5 μg of Alexa647-labeled CD19 probe. Five hours after probe injection, blood, bone marrow, and spleen samples were collected for flow cytometric analysis (Fig. 2A). Gating on green fluorescent protein–positive (GFP+) CAR T cells in each respective organ revealed robust Alexa647 labeling in CD19 CAR T cells (91.5% in blood, 94.5% in bone marrow, and 83.3% in spleen) but undetectable staining in either control CAR T cells or non–CAR T cell populations (Fig. 2, B and C). Moreover, the mean fluorescence intensity (MFI) of Alexa647-CD19 probe binding to CD19 CAR T cells was consistently high in all analyzed organs (Fig. 2D). Collectively, the results affirm the CD19 probe’s high affinity and specific binding to the CD19 CAR T cells, both in vitro and in vivo*.*

### Zr-labeled PEGylated CD19 imaging probe retains capacity to bind to CD19 CAR T cells with high affinity

We next assessed whether the inclusion of the chelator deferoxamine (DFO) and zirconium ions would affect the binding characteristics of the CD19 probe to CD19 CAR T cells. We opted to use ^89^Zirconium (^89^Zr) as the PET isotope because of its broad clinical applicability and a half-life of approximately 3.3 days, allowing for imaging several hours after the probe injection. In addition, a 20-kDa polyethylene glycol (PEG) moiety was installed at the C terminus to improve image quality (*68*, *69*). CD19 CAR T cells and control CAR T cells underwent incubation with the CD19 probe, which had been site-specifically labeled with DFO chelator and a PEG moiety (20 kDa in size) using a sortase reaction, following our previously established protocol (*68*, *69*). We assessed both CD19 probe labeled with PEG and DFO alone (DFO-PEG-CD19) and CD19 labeled with PEG and DFO installed with a zirconium ion (Zr-DFO-PEG-CD19). The concentrations ranged from 0 to 100 nM. For detection of the probe, we used an anti-FLAG secondary antibody. The results demonstrated that both DFO-PEG-CD19 and Zr-DFO-PEG-CD19 (fig. S1, C and D) displayed specific binding to CD19 CAR T cells, with no binding to control CAR T cells. In addition, both constructs exhibited high binding affinity to CD19 CAR T cells with low nanomolar affinity. While the Zr-DFO-PEG-CD19 probe’s observed binding affinity was lower compared to that achieved with the Alexa647-CD19 probe (EC_50_ 0.074 nM for Alexa647-CD19 versus 0.96 nM for DFO-PEG-CD19 and 1.07 nM for Zr-DFO-PEG-CD19), it still falls within the low nanomolar range, indicating that the probe’s binding affinity remains suitable for in vivo imaging. To confirm the probe’s specificity in binding to human CAR T cells, we stained freshly prepared human CD19 CAR T cells in the presence or absence of human peripheral blood mononuclear cells (PBMCs). Results demonstrated that the probe exclusively binds to CD19 CAR T cells and not to any other cell populations within the PBMCs (fig. S1, E and F).

### The CD19 probe does not affect functionality of CAR T cells

**Fig. 3.**
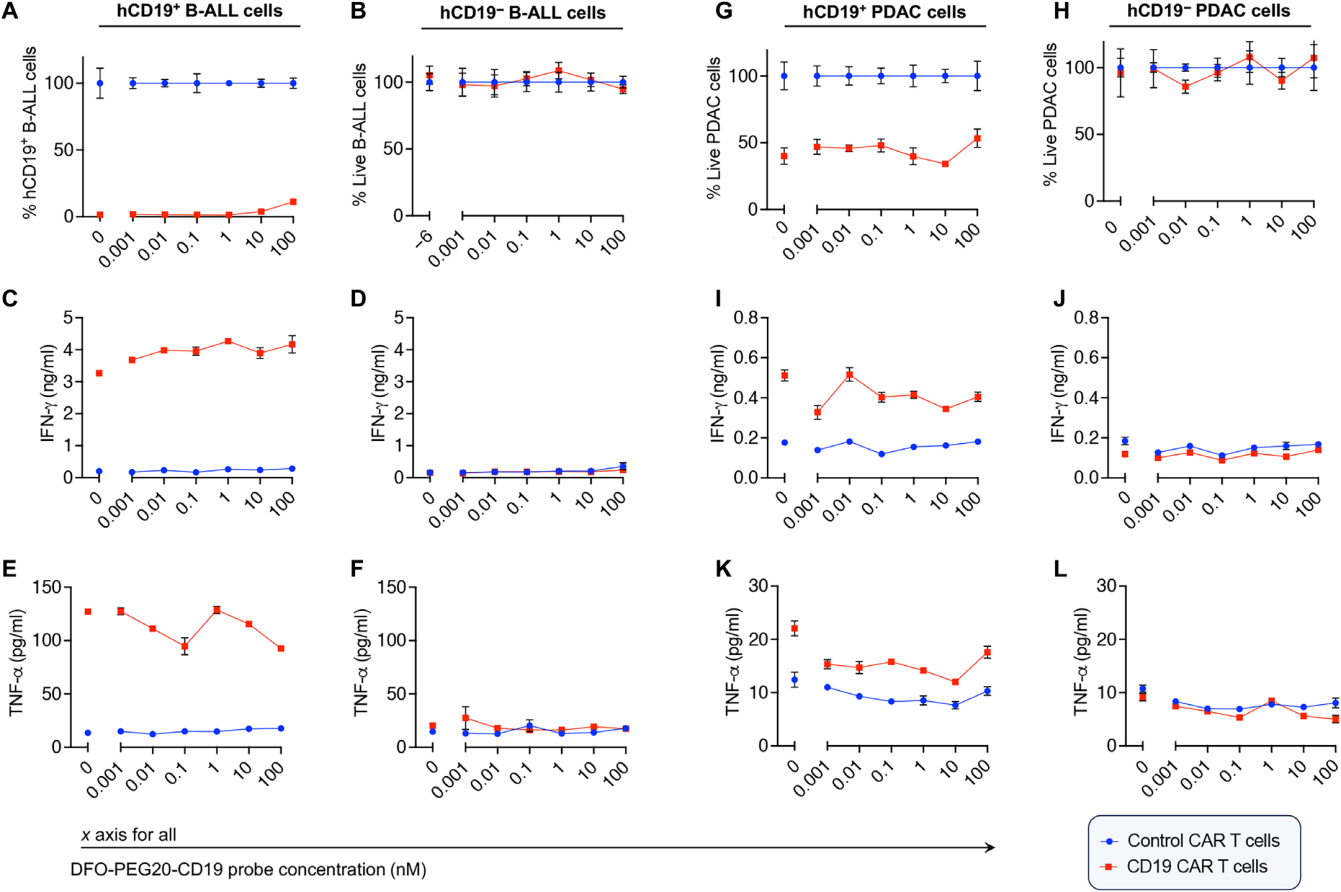
Evaluation of the impact of the CD19 probe on CAR T cell activity. (**A** to **F**) CAR T cell cytotoxicity assay using CD19 CAR T or irrelevant CAR T with B-ALL cells (E-T ratio = 10:1) with and without the hCD19 antigen, in the presence of varying doses of the DFO-PEG20-CD19 probe. (A) Percentage of hCD19^+^ B-ALL cells at 24 hours was determined to assess antigen loss. (B) Viability of hCD19- B-ALL cells at 24 hours confirming no nonspecific killing. (C and D) IFN-γ release by CD19 or control CAR T cells in the presence of B-ALL cells expressing (C) and not expressing (D) the hCD19 antigen. (E and F) TNF-α release by CD19 or control CAR T cells in the presence of B-ALL cells expressing (E) and not expressing (F) the hCD19 antigen in the presence of the CD19 probe. (**G** and **H**) CAR T cell cytotoxicity assay using CD19 CAR T or irrelevant CAR T with PDAC cells (E-T ratio = 10:1) with and without the hCD19 antigen, in the presence of varying doses of the CD19 probe. Tumor cell counts were determined 24 hours later. (**I** and **J**) IFN-γ release by CD19 or control CAR T cells using different concentrations of the probe in the presence of PDAC cells expressing (I) and not expressing (J) the hCD19 antigen. (**K** and **L**) TNF-α release by CD19 or control CAR T cells using different doses of the CD19 probe, in the presence of PDAC cells expressing (K) and not expressing (L) the hCD19 antigen. All data analyzed in triplicates, and error bars represent SEM. Murine T cells expressing anti-human CD19 with a murine CD28-CD3z construct were used as CD19 CAR T cells. B-ALL and PDAC cancer cells were murine cells expressing the human CD19 antigen or not, as specified. *X* axes represent CD19 probe (DFO-PEG20-CD19) concentrations in nanomolar. *N* = 3 technical replicates were performed for each condition.

Our next objective was to investigate whether the CD19 probe binding had any impact on CD19 CAR T cell functionality. We first assessed whether the interaction between the CD19 probe and CD19 CAR on T cells impeded CAR T cell killing activity in vitro. To do this, we conducted experiments in which CAR T cells were cocultured with either murine B-ALL cells (hCD19^+^ B-ALL) or murine pancreatic cancer cells engineered to express the ectodomain of human CD19 [hCD19^+^ Pancreatic Ductal Adenocarcinoma (PDAC)]. Murine CD19^+^ B-ALL cells are typically less susceptible to CAR T cell–mediated killing in vitro due to the rapid endocytosis of the target antigen; thus, hCD19^+^ PDAC cells were included as an additional experimental condition (*66*). To perform these cytotoxicity assays, B-ALL or PDAC cells were incubated with either CD19 or control CAR T cells at an effector-to-target (E:T) ratio of 10:1 with increasing concentrations of the DFO-PEG20-CD19 probe (ranging from 0 to 100 nM). After 24 hours, end point data were evaluated with flow cytometry. Given the decreased susceptibility to killing observed in hCD19^+^ B-ALL cells in vitro, surface levels of hCD19 on B-ALL cells as well as release of IFN-γ and TNF-α by CAR T cells in the assay were assessed as indicators of CAR T cell function (Fig. 3, A and F, and fig. S2A). For hCD19^+^ PDAC cells, cell viability, interferon-γ (IFN-γ) and tumor necrosis factor–α (TNF-α) release were evaluated to assess CAR T cell function (Fig. 3, G to L, and fig. S2B).

Of note, the CD19 probe was not removed during the killing assays and remained bound to CAR T cells in a dose-dependent manner at the time of analysis (fig. S3, A to D). The presence of the CD19 probe at concentrations of 1 nM or lower did not affect antigen loss on hCD19^+^ B-ALL cells (Fig. 3A). A slight increase in antigen retention on B-ALL cells was only observed when the probe concentration reached 10 nM or more (Fig. 3A and fig. S2A). Furthermore, when evaluating the levels of IFN-γ or TNF-α released by CD19 CAR T cells or control CAR T cells, we did not observe a significant reduction in IFN-γ (Fig. 3C) or TNF-α production (Fig. 3E) from CD19 CAR T cells when they were cocultured with hCD19^+^ B-ALL cells. The CD19 probe did not hinder the killing of hCD19^+^ PDAC cells, even at probe concentrations as high as 100 nM (Fig. 3G). Our analysis also revealed that CD19 CAR T cells did not exhibit reduced IFN-γ release (Fig. 3I) or TNF-α (Fig. 3K) production, even when the probe concentration reached 100 nM in the presence of hCD19^+^ PDAC cells. These results suggest that the probe does not hinder CAR T cell killing activity. The dominance of membrane-bound CD19 antigen, facilitated by synapse formation between CAR and tumor cells, likely leads to higher avidity interactions with CAR molecules, preventing interference from soluble CD19 antigen binding. Similar killing assays were performed using human CAR T cells and human Nalm6 cancer cells (E-T ratios of 1:1 and 1:10), which further supported these findings: Even at concentrations of 200 nM, the CD19 PET probe did not impair cytotoxicity of human CAR T cells (fig. S4). Of note, in an in vivo experiment, the probe will be rapidly cleared from the circulation, further minimizing interference with CAR T activity.

We also assessed whether the binding of the CD19 probe to CD19 CAR T cells would activate the CAR T cells and lead to potential nonspecific killing of antigen-negative cells. Accordingly, we performed assays in which antigen-negative B-ALL or PDAC cells were combined with CD19 CAR T and control CAR T cells, followed by the addition of increasing concentrations of the CD19 probe. We observed no dose-dependent killing of the antigen-negative B-ALL cells (Fig. 3B). There was no significant release of IFN-γ or TNF-α, and the levels of IFN-γ and TNF-α released remained consistent regardless of the quantity of CD19 probe added to the killing assay (Fig. 3, D and F). This demonstrated that only interaction with antigen-positive B-ALL cells, not the CD19 probe, could stimulate IFN-γ release from the CD19 CAR T cells. Similarly, when CD19 CAR T cells were mixed with antigen-negative PDAC cells and varying amounts of the probe, we observed no dose-dependent killing (Fig. 3H), IFN-γ release (Fig. 3J), or TNF-α release (Fig. 3L).

To replicate the complex conditions encountered by CAR T cells in vivo, we next investigated whether administering the CD19 probe would have any impact on CAR T cell function in a syngeneic model. We first established that in vivo hCD19^+^ B-ALL tumor burden is significantly reduced with treatment with CD19 CAR T cells but not control antigen (EGFRvIII)–targeting CAR T cells (fig. S5, A and B). Next, we transplanted mice that had been previously sublethally irradiated (1 × 5 Gy) with 3 × 10^6^ hCD19^+^ B-ALL cells and administered CAR T cells targeting human CD19 or control antigen (EGFRvIII) 3 days later. While radiation is not necessary for tumor cell engraftment in this syngeneic model, it does mirror the pretreatment priming received by patients before CAR T engraftment. Following 2 days of CAR T cell treatment, we injected 5 μg of the CD19 probe into the mice. This CD19 probe was installed with a PEG moiety, labeled with the DFO and loaded with a Zr ion, mirroring the PET probe that will be used for PET imaging (fig. S5C). Treatment with CD19 CAR T cells significantly prolonged the survival of leukemic mice compared to those treated with control CAR T cells (fig. S5D). There was no disparity in survival between mice treated with CD19 CAR T cells with or without the CD19 probe. Therefore, these findings suggest that the current administered dose of the CD19 probe does not interfere with the functional activity of CD19 CAR T cells in vivo*.*

### Radiolabeled CD19 probe detects CD19 CAR T cells in vivo with high specificity

Having established that the site-specific Zr-DFO labeling preserves the probe’s binding affinity and specificity, we performed PET imaging to visualize CD19 CAR T cells in vivo. To facilitate this, we conjugated the DFO-CD19 probe with radioactive ^89^Zr ions using established protocols, resulting in >99% purity as confirmed by radio thin-layer chromatography (radio-TLC) analysis (fig. S6). The ^89^Zr-labeled probe was then administered to C57BL/6 mice that had previously received transplants of B-ALL cells and had been treated with either CD19 or control CAR T cells (fig. S7A). To improve in vivo image quality, minimize kidney uptake, and enhance specific uptake, we conducted a comparison between CD19 probes linked to different lengths of PEG (5, 10, and 20 kDa). The results demonstrated that mice treated with CD19 CAR T cells exhibited a specific PET signal in the spleen and bone marrow visualized on in vivo images and confirmed on ex vivo images (fig. S7, B and C), suggesting that the probe can detect CAR T cells in vivo with clarity. This analysis further revealed that the installation of the PEG20 molecule onto the CD19 probe resulted in the least kidney retention, most improved signal-to-noise ratio, and best PET image quality (fig. S7, B and C). This outcome aligned with our previous finding that incorporating PEG groups reduces kidney retention of PET probes while enhancing specific uptake (*68*, *69*). Of note, adding PEG molecules increases the probe’s circulation half-life (*68*). To determine the optimal imaging time point after injection of the ^89^Zr-DFO-PEG20-CD19 probe, the mice were serially imaged at 24, 48, and 96 hours. Imaging at 24 hours showed comparable signal-to-noise ratio to 48 and 96 hours (fig. S7, D and E), indicating that next-day imaging is feasible. The clearance of the probe from the blood within 48 hours after injection was also evaluated—revealing rapid elimination with clearance of approximately half of the probe within the first 2 hours (fig. S7F). Consequently, subsequent experiments were conducted using the CD19 probe with the site-specific installation of a PEG20 with imaging at 24 hours.

We next evaluated the serum stability of the probe installed with PEG20. Briefly, the DFO-PEG20-CD19 probe was added into freshly collected murine serum from C57BL/6 mice, reaching a final concentration of 1 μM. Subsequently, the mixture was divided into two fractions and stored at 4° or 37°C. Samples were obtained at various time intervals (6, 24, 48, and 72 hours) and promptly flash-frozen in liquid nitrogen. Upon collection of all samples, their binding to CD19 CAR T cells was assessed. Even after incubation for 3 days at 37°C in the serum, the probe maintained its efficacy in binding to CD19 CAR T cells within the low nanomolar range, with only a marginal decrease in binding observed (fig. S8). Given that the time for PET acquisition is 24 hours after probe injection, the stability of the CD19 probe exceeds the necessary requirements.

**Fig. 4.**
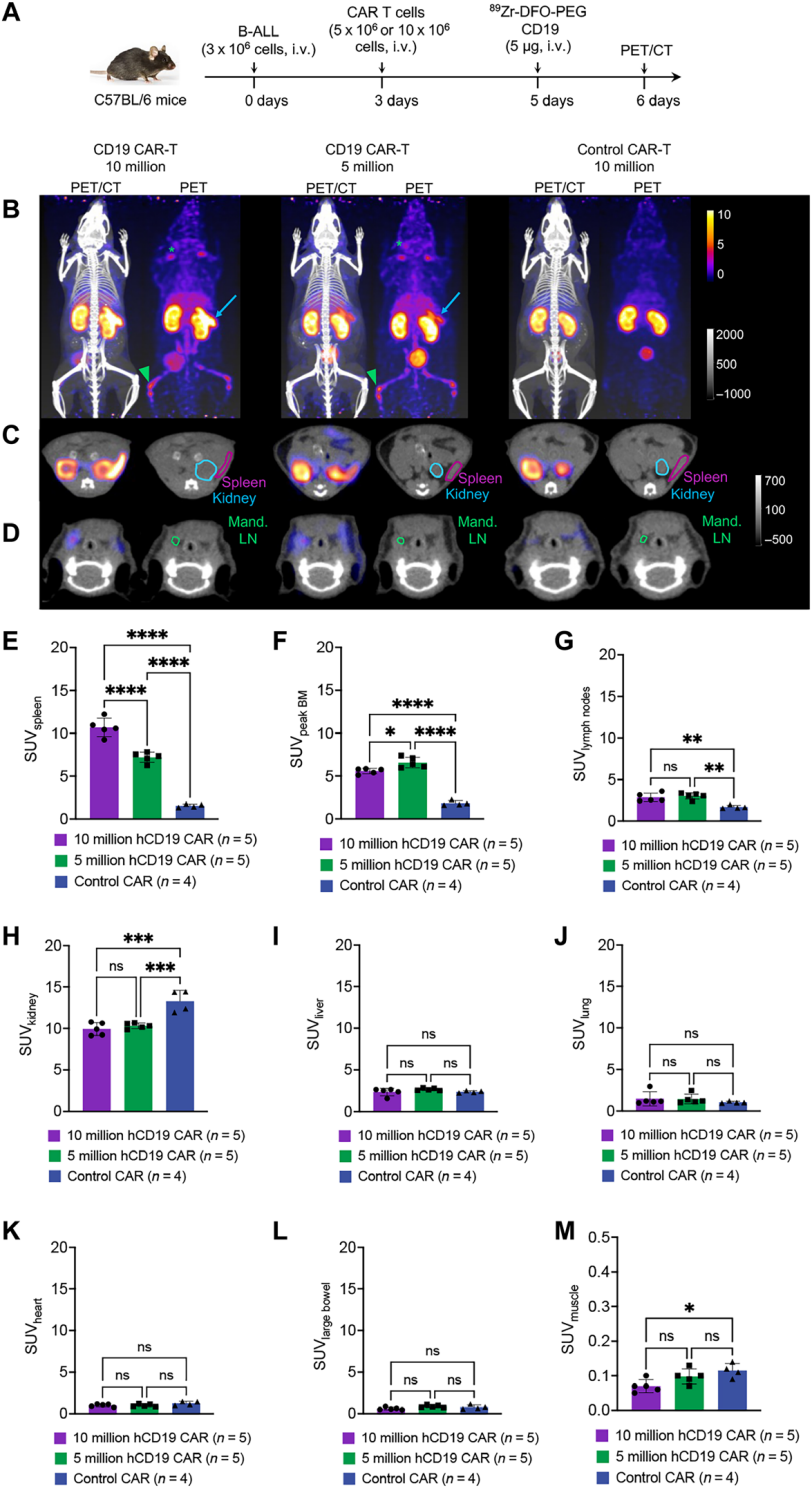
The CD19 PET probe detects CD19 CAR T cells in vivo in a syngeneic mouse model of B-ALL and can differentiate between different doses of CAR T cells. (**A**) Experimental schematic: C57BL/6 mice received an intravenous injection of 3 million murine B-ALL cancer cells expressing human CD19, followed by a subsequent intravenous injection of freshly prepared CD19 CAR T cells (expressing anti-human CD19 with a murine CD28-CD3ζ endodomain CAR construct, dose of 5 million or 10 million CAR T cells; *n* = 5 for each cohort) or control CAR T cells (anti-EGFR, dose of 10 million CAR T cells; *n* = 4) three days later. Two days after CAR T cell injection, the mice were administered the ^89^Zr-DFO-PEG20 CD19 probe (5 μg, ~50 μCi, intravenous). (**B**) PET-CT imaging of mice 24 hours after probe injection. Left, whole-body CT scan with PET signal overlay. Right, PET image alone. Injected CAR T cell quantities are shown above the images. Arrows indicate spleen, triangles denote the area of the highest bone marrow in the skeleton (peak bone marrow), and stars mark lymph nodes in PET images. See movies S1 to S3. (**C** and **D**) Transverse views of mice in PET-CT overlay (left) and CT only (right) at 24 hours after CD19 probe injection, showing (C) kidney and spleen and (D) mandibular lymph nodes in CT images. Images shown in (B) to (D) are representative of *n* = 4 to 5 for each group with similar results. (**E** to **M**) Quantification of mean standardized uptake values (SUV_mean_) of CD19 probe in (E) spleen, (F) peak bone marrow, (G) lymph nodes, (H) kidney, (I) liver, (J) lung, (K) heart, (L) large bowel, and (M) muscle 24 hours after probe injection. Error bars represent SD. Statistical analysis was performed using ordinary one-way analysis of variance (ANOVA) (ns > 0.05, * ≤ 0.05, **P ≤ 0.01, ***P ≤ 0.001, ****P ≤ 0.0001). Mand. LN, mandibular lymph node; BM, bone marrow.

Following our optimization of imaging quality and further confirming its serum stability, we conducted imaging on a larger group of mice using the ^89^Zr-DFO-PEG20-labeled CD19 probe. In this experimental setup, mice were intravenously injected with 3 million hCD19^+^ B-ALL cells, followed by the administration of 10 million CD19, or control (EGFRvIII) CAR T cells 3 days later (Fig. 4A). In addition, a third group received 5 million CD19 CAR T cells to assess the probe’s ability to differentiate between different CAR T cell doses. Two days after CAR T cell injection, the ^89^Zr-DFO-PEG20–labeled CD19 probe was intravenously administered (~50 μCi of activity, ~5 μg of the probe, per mouse). Twenty-four hours after injection of the ^89^Zr-DFO-PEG20–labeled CD19 probe, we subjected the animals to PET imaging with concurrent computed tomography (CT) imaging to localize PET signal to anatomic structures (Fig. 4A). The mean standard uptake values (SUV_mean_) of the probe in vivo were quantified for tissues of interest in each mouse. To account for the variability in CAR T cell homing within the bone marrow across different mice, the SUV_mean_ of the region with the highest bone marrow signal (“peak bone marrow”) was measured. This value was used as an approximation of the total bone marrow CAR T cell population for each mouse, with a consistent measurement volume of approximately 0.75 mm^3^ across all mice. The resulting PET images visualize distinct signal in the spleen and bone marrow of mice injected with CD19 CAR T cells, which are the primary sites of leukemic burden. In contrast, these signals were absent in mice administered with control CAR T cells (Fig. 4, B and C, and movies S1 to S3). Mice that received a higher dose of 10 million CD19 CAR T cells exhibited a higher PET signal in the spleen compared to the group receiving 5 million CD19 CAR T cells, indicating the method’s ability to differentiate between lower and higher doses of CAR T cells (Fig. 4E). However, the signal in the bone marrow was similar between the two groups, both of which were significantly higher than the control groups (Fig. 4F). The probe could also detect lymph nodes, suggesting the approach’s capability to reliably detect even low numbers of CAR T cells (Fig. 4, D and G). To evaluate the biodistribution of the CD19 probe beyond tissues with leukemic burden, the SUV_mean_ was quantified for additional organs, including the kidneys, lungs, heart, liver, large bowel, and muscle (Fig. 4, E to M).

**Fig. 5.**
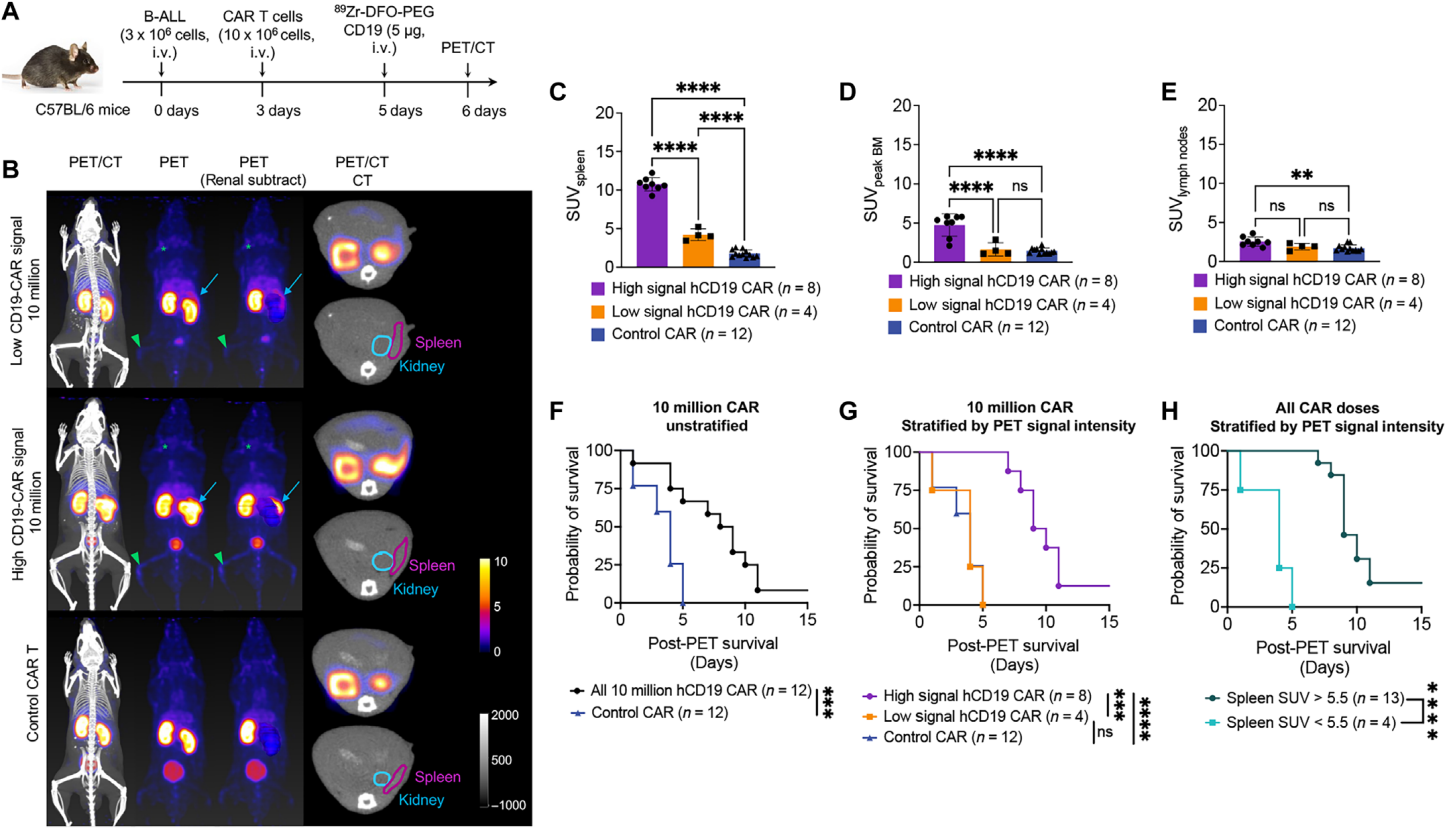
The CD19 CAR-PET signal intensity predicts early mortality risk. (**A**) Mice received human-CD19^+^ B-ALL cells, followed by either CD19 (*n* = 7) or control (*n* = 8) CAR T cells. Subsequently, they underwent PET-CT imaging according to the schedule. (**B**) PET-CT images. Left to right, whole-body CT scan overlaid with PET, PET alone, PET with ipsilateral renal signal subtracted to allow improved spleen visualization, and a transverse view of the mouse in PET-CT overlay and CT only at 24 hours after CD19 probe injection. Arrows indicate spleen location in PET images, and circles denote the kidney (blue) and spleen (purple) in transverse CT images. The mice exhibited heterogeneous CAR-PET signal. Representative images with low CAR-PET signal are shown at the top, high signal in the middle, and control mice at the bottom. See movies S4 to S9. (**C** to **E**) Quantification of probe SUVmean in the (C) spleen, (D) area of highest bone marrow in the skeleton (peak bone marrow), and (E) lymph nodes 24 hours after probe injection. The mice were divided into high and low CD19-PET signal groups based on spleen SUVs (below or above 5.5). Data from mice that received 10 million CD19 (*n* = 5) or control CAR T (*n* = 4) shown in Fig. 4 are included for comprehensive analysis (total *n* = 12 for each group). Error bars represent SD. Statistical analysis conducted using ordinary one-way ANOVA. (**F** and **G**) Survival analysis of mice, independent of SUV values [(F) Mantel-Cox *P* = 0.0008] and stratified on the basis of high (purple) and low (orange) spleen SUV values (using SUV of 5.5 as a threshold) [(G) Mantel-Cox *P* = 0.003 high versus low signal mice, *P* < 0.0001 high signal versus control mice]. (**H**) Pooled survival analysis of mice treated with 10 or 5 million CD19 CAR T cells segmented by spleen SUV values (using SUV of 5.5 as a threshold). Mantel-Cox *P* < 0.0001. ns > 0.05, *P ≤ 0.05, **P ≤ 0.01, ***P ≤ 0.001, ****P ≤ 0.0001.

We repeated this study with the same experimental setup with the exception that all mice received 10 million CAR T cells (Fig. 5A). Quantitative analysis of the resulting PET images revealed two distinct groups of the CD19 CAR T–treated mice, where one group displayed intense PET signal in lymphoid organs and the other displayed much lower (although still above background) PET signal in the same organs (Fig. 5B, fig. S9, and movies S4 to S9). We pooled the results of this experiment with those mice given 10 million CAR T cells presented in Fig. 4 and segmented mice into two groups: those with “high CD19-PET signal” (spleen SUV_mean_ > 5.5, *n* = 8 with mean 10.74 ± 0.86) and those with “low CD19-PET signal” (spleen SUV_mean_ < 5.5, *n* = 4 with mean 4.21 ± 0.74). In contrast, the mean spleen SUV_mean_ for mice injected with control CAR T cells was approximately 1.77 ± 0.47 (Fig. 5C). Of note, low signal mice had an SUV_mean_ range of 3.45 to 5.24; thus, a cutoff of 5.5 SUV_mean_ was chosen to denote mice with low spleen PET signal for downstream analyses.

Bone marrow signal largely mirrored the PET signal intensity seen in the spleen, where mice with high spleen signal also had significantly higher bone marrow signal (mean 4.75 ± 1.41) compared to low signal (mean 1.65 ± 0.83) and control mice (mean 1.46 ± 0.36) (Fig. 5D). Only the high CD19-PET signal mice showed a significant difference in lymph node signal compared to control (Fig. 5E).

We closely monitored the survival of these mice during the PET imaging period. Notably, we observed an extension in survival among the mice in the high CD19-PET signal group compared to those in the low CD19-PET signal group (Fig. 5, F and G). The CD19 CAR T–treated mice in the low CD19-PET signal group exhibited similar survival rates to those treated with control CAR T cells (Fig. 5G). When survival analyses were pooled across both 5 and 10 million CAR T cell doses, we observed that all mice with spleen signals >5.5 SUV_mean_ survived past 6 days after PET imaging, while all the mice with spleen signals <5.5 SUV_mean_ died by day 5 after PET imaging (Fig. 5H). Overall, CAR-PET signal in the spleen stratified mice by risk of early mortality.

In summary, our findings demonstrate that the antigen-based CD19 PET probe can detect CD19 CAR T cells in vivo with high specificity and sensitivity, including in organs like the spleen, bone marrow, and lymph nodes of leukemic mice treated with CD19 CAR T cells. The probe rapidly clears from the bloodstream, allowing for next-day imaging. The strength of the PET signal 24 hours after the probe injection correlates with the response to CD19 CAR T cell treatment in a murine model of B-ALL.

## DISCUSSION

CAR T cell therapy displays heterogeneous responses and unpredictable relapse rates. Current nonimaging methods lack crucial temporospatial information on CAR T cell populations in vivo, hindering comprehensive evaluation. Peripheral blood monitoring provides limited insights, and crucial information on CAR T cell viability and presence in major organs, lymphoid structures, and bone marrow remains largely unknown. These insights are critical for treatment response assessment, relapse prediction, and understanding adverse outcomes like cytopenias. PET, a whole-body imaging technique, has the potential to address these questions, offering a comprehensive perspective on CAR T cell dynamics. While several reporter gene–based CAR-PET imaging approaches exist, all require additional engineering steps on CAR T cells to include a reporter gene. The ex vivo–labeling approach does not require genetic modification but lacks the flexibility to image at chosen time points, limiting its clinical applicability.

To overcome existing CAR-PET imaging limitations, we introduce an “antigen-based” CAR-PET imaging approach using the ectodomain of the human CD19 protein as a PET probe. In vitro, the PET probe demonstrated high binding affinity with EC_50_ in the low nanomolar range—well within the required blood concentration for PET imaging agents (*70*, *71*). Further, in vitro and in vivo analyses also highlighted the specificity of the CD19 probe in binding to CD19 CAR T cells, even in the presence of human PBMCs. This can be attributed to the exceptionally high specificity and affinity of antigen-antibody interactions. Furthermore, the CD19 PET probe has no adverse impact on CD19 CAR T cell anticancer functionality, especially in the low nanomolar range, which is the in vivo working range for the PET probe. The probe also demonstrates a favorable safety profile: We observed no activation of CD19 CAR T cells or nonspecific killing of CD19^−^ cells, even in the presence of high concentrations. These results indicate that the probe does not compromise the killing efficacy of CAR T cells, consistent with clinical studies showing that soluble antigen levels do not significantly affect CAR T therapy efficacy (*3*, *72*–*74*). Similarly, an antigen-based BCMA CAR-enhancer has been shown to improve the activity and persistence of BCMA CAR T cells in preclinical models, without reducing their cytotoxic efficacy (*75*). This lack of interference may be attributed to the dominance of membrane-bound antigen binding to CAR T cells, facilitated by synapse formation between CAR and tumor cells. This dynamic results in heightened avidity compared to monomeric soluble antigen binding to a CAR molecule. However, it is important to study how different antigens may affect the activity of CAR T cells, especially in tumors with heterogeneous or low antigen density, where competition for CAR binding may become a considerable factor. In addition, the probe will be rapidly cleared from the bloodstream within a few hours (fig. S7F), providing further assurance that it will not interfere with the cytotoxic activity of CAR T cells. Furthermore, the probe’s monomeric nature avoids inducing IFN-γ or TNF-α production in CAR T cells, likely because it does not trigger clustering of the CAR molecule (*76*). Therefore, the antigen PET probe is expected to exhibit a high safety profile when translated into a clinical setting, without adversely affecting CAR T cell activity.

In vivo imaging with the CD19 probe in a syngeneic model of B-ALL demonstrated high specificity with minimal background uptake, primarily limited to kidney retention, comparable to leading reporter gene strategies for imaging CAR T cells (*51*). Adding a 20-kDa PEG group to our CD19 probe improved specific uptake and reduced nonspecific kidney retention. It is worth mentioning that while kidney retention of the probe in mice may complicate spleen uptake analyses, the distinct anatomical separation between the kidneys and spleen in humans suggests that assessing spleen uptake would not be affected. While PEGylation with PEG20 of the antigen-based probe does prolong its circulatory half-life, its size of ~52 kDa still falls below glomerular filtration size of ~60 kDa. This preserves the probe’s rapid clearance within a few hours of injection and facilitates next-day imaging (fig. S7).

The in vivo images demonstrate the probe’s reliable detection of CD19 CAR T cells, visualizing even small populations in the bone marrow and lymph nodes (Figs. 4 and 5). This suggests that the CD19 probe is highly sensitive in detecting CAR T cells; however, additional studies are needed to precisely and quantitatively evaluate the approach’s sensitivity and compare it with existing reporter gene strategies. Mice injected with varying CAR T doses exhibited spleen signals correlating with the injected CAR T quantity, with higher signal intensity in mice receiving 10 million CAR T compared to those receiving 5 million CAR T (Fig. 4E). However, both groups displayed similar CAR T signals in their bone marrow and lymph nodes, with even the 5 million group showing slightly higher signal in the bone marrow (Fig. 4, F and G). This highlights the complex distribution of CAR T cells across organs and emphasizes that CAR T homing and in vivo expansion are influenced not only by the initial CAR T quantity but also by the specific organ. CAR T expansion may be more pronounced in organs like the spleen because of available space, while being constrained in organs like the bone marrow. Such insights can be particularly valuable clinically, as detecting CAR T in the bone marrow would be challenging with methods other than PET. Further, we observe that spleen CAR-PET signal intensity could be used to stratify mice by risk of early mortality. For example, 100% of mice with spleen signals >5.5 SUV_mean_ survived past 6 days after PET imaging, while 100% of mice with spleen signals <5.5 SUV_mean_ died by day 5 after PET imaging, reinforcing the clinical relevance of this imaging approach. CAR T signal intensity in organs such as spleen and bone marrow could provide valuable information that can offer insights into the treatment response and chance of relapse. However, while proliferation and persistence in lymphoid organs may serve as a reliable surrogate for response in blood-borne malignancies, this may not necessarily apply to solid tumors, where factors such as tumor infiltration and penetration may be more critical. Future studies could investigate whether the PET probe can effectively detect CAR T cell infiltration into tumors and how this correlates with therapeutic response.

CAR T cell imaging with PET represents a powerful tool for accurately visualizing post-infusion CAR T cell presence in patients. While several CAR imaging strategies already exist, there are several advantages to the antigen-based PET strategy presented here. Unlike the reporter gene strategy, there is no requirement to reengineer CAR T cells. Thus, this method is applicable to both FDA-approved CAR T cells and newer generations of CAR cells in the development pipeline. The probe, being a self-protein, is anticipated to have low immunogenicity, allowing for serial imaging if necessary. Real-time imaging of CAR T cells at any desired time point is achievable, as patients can be imaged in a longitudinal fashion, enabling one to view both CAR T dynamics during tumor remission and long-term CAR T persistence that affects the regeneration of the normal B cell compartment. One notable advantage of the reporter-gene strategy is that imaging can be done the same day, as the small molecules used as PET tracers are typically rapidly cleared from circulation. However, while the CD19 probe presented here optimally performed at 24 hours after probe injection, this is still a practical imaging schedule that is already used in clinical settings (*24*, *77*).

One major challenge in the CAR T therapy arena is the unpredictability of patient relapse. While CAR T cell therapies have achieved remarkable outcomes for B cell malignancies, an estimated 40 to 60% of patients with complete response will eventually relapse (*1*, *10*, *12*, *16*). Several studies have shown that patients with higher peak blood CAR levels had longer duration of response for B cell malignancies (*78*–*82*). However, blood CAR levels provide no information about the spatial distribution of CAR T cells in the body after infusion, which can provide important and more accurate insights for a patient’s likelihood and durability of response. For example, it is known that the tumor microenvironment (TME) in B cell malignancies can affect CAR T cell efficacy (*83*). CAR T cell trafficking into certain tissues with cancer burden, such as bone marrow, central nervous system, and primary solid tumors, can be hindered by the presence of chemokines such as CXCL12 (*83*). Further, the TME of these tissues can feature hostile metabolic environments for CAR T cells, characterized by hypoxia and nutrient scarcity (*84*, *85*). This milieu is thought to impair the proliferation and cytotoxicity of CAR T cells. Thus, current methods like peak blood CAR levels cannot ascertain whether CAR have successfully reached and expanded within particularly challenging cancer sites, which growing evidence suggests is a major factor in predicting risk of relapse (*81*, *83*, *86*). Another key driver of relapse risk is lack of CAR T cell persistence (*1*). However, identifying patients with CAR persistence is hampered by the rapid disappearance of CAR T cells from the circulation that typically occurs within 1 to 3 months after administration, which makes longitudinal monitoring of CAR T cell populations with blood levels alone challenging (*82*). Therefore, in contrast with current widely used methods for detecting CAR T cells, CAR-PET imaging would provide an important temporospatial dimension to monitor CAR T cells in vivo, with potentially impactful implications for predicting relapse. Moreover, this may also be used to refine and maximize the precision and efficacy of the therapy, particularly, as new strategies for improving CAR homing and persistence are being developed (*87*).

While CAR-PET imaging may improve our ability to predict relapse, it may also shed light on who is most at risk of adverse effects from CAR T cell therapy. For example, cytopenias are a common side effect of CAR T cell therapy, with increased morbidity and mortality due to infection and bleeding risks depending on the affected cell type (*88*–*90*). While most cytopenias are likely secondary to lymph depletion therapy and, therefore, self-resolve within 30 days after CAR T cell infusion, a portion of patients will experience prolonged or late-onset cytopenias. The underlying biology of these cytopenias remains elusive but may be driven in part to CAR T cell persistence and toxicity in bone marrow (*88*).

Expanding upon this strategy, PET probes similar to the CD19 probe developed here can be developed for additional CARs targeting distinct protein tumor antigens, such as BCMA, CD22, and HER2. The clinical integration of antigen-based PET probes for CAR T cell imaging offers a seamless pathway. This approach maintains a high level of specificity in CAR imaging. As previously discussed, the global adoption of CAR T therapies continues to expand, encompassing diverse indications, spanning from cancer to autoimmune diseases. The antigen-based CAR-PET probe methodology, requiring no engineering of CAR T cells, facilitates real-time imaging of CAR T cells, providing valuable insights for managing patients undergoing CAR T therapy.

## MATERIALS AND METHODS

### Cell culture and cell lines

The cell lines used in this study comprised murine B-ALL cells (*66*), murine PDAC cells (provided by the Vander Heiden Laboratory), and HEK293T. HEK293T cells, acquired from the American Type Culture Collection, were sustained in Dulbecco’s modified Eagle’s medium (DMEM) with l-glutamine and sodium pyruvate (Corning, 10-013-CM) supplemented with 15% fetal bovine serum (FBS). Murine B-ALL cells were cultured in RPMI 1640 with l-glutamine (Corning, 10-040-CM) supplemented with 10% FBS and 2-mercaptoethanol to a final concentration of 0.05 mM (Gibco, 21985023). Murine PDAC cells were grown in DMEM with l-glutamine and sodium pyruvate (Corning, 10-013-CM) supplemented with 10% FBS. Routine mycoplasma contamination checks were conducted for all cell lines.

Primary murine T cells were procured from mouse spleens and cultured on plates coated with activating antibodies, as described in the CAR T cell production methods. The T cell medium consisted of RPMI 1640 with l-glutamine (Corning, 10-040-CM) supplemented with 10% FBS, recombinant human IL-2 (rhIL-2, final concentration of 20 ng/ml; Peprotech, catalog no. 200-02-1mg), and 2-mercaptoethanol to a final concentration of 0.05 mM (Gibco, 21985023).

### Viral supernatant production

Viral supernatant was produced using standard methods. Briefly, HEK293T cells were transfected with retroviral or lentiviral transfer plasmid and packaging vector (retrovirus: pCL-Eco, Addgene, 12371; lentivirus: psPAX2, Addgene, 12260 with VSVg envelop plasmid pMD2.G, Addgene, 12259) using Mirus TransIT-LT1 (Mirus, MIR2305) as indicated by the manufacturer. Viral supernatant was collected 48 and 72 hours after transfection, passed through a 0.45-μm filter, and stored at 4°C for a maximum of 1 day.

### Probe production

All genes underwent codon optimization for HEK293T mammalian expression, followed by synthesis and insertion into a vector expression system equipped with a signal sequence. A stable HEK293T cell line was established through transfection with pPAX2, pVSVG (packaging vectors), and the lentivirus plasmid containing the sequence of interest. Virus harvesting occurred at 48, 72, and 96 hours, followed by sedimentation at 20,000*g* for 2 hours and resuspension in optiMEM media. Fresh HEK293T cells were then transduced with the virus and allowed to recover in complete DMEM. Puromycin selection was applied to retain cells that integrated the lentivirus plasmid. After expansion, cells transitioned to serum-free media for 48 to 72 hours before commencing supernatant harvesting.

After collection, protein expression in the supernatant was validated via SDS-PAGE. Subsequently, proteins underwent purification using a Ni–nitrilotriacetic acid (NTA) metal affinity column. Nonspecifically bound proteins were eliminated through washing with a low-concentration imidazole solution (40 mM), and the protein of interest was recovered using a high-concentration imidazole solution (400 mM). The final purification step involved size exclusion chromatography, and the purified proteins were stored in 50 mM Hepes buffer, (pH 7.5), at −80°C until use.

The DFO-labeled CD19 with PEG 20 kDa was prepared following our established procedure (*68*). For DFO labeling, 1 liter of 50 mM Hepes with 150 mM NaCl (pH 7.5) underwent chelex treatment for 16 hours at 4°C using 10 g of Chelex 100 sodium form beads to eliminate trace metals. After chelexing, the protein and all reagents, including sortase, were dialyzed in trace metal–free Hepes using Thermo Fisher Scientific SnakeSkin Dialysis Tubing for an additional 16 hours. After dialysis, GGG-azide-DFO substrate, dissolved in dimethyl sulfoxide was added to the protein at a concentration of 2 mM. Subsequently, Sortase 7M was included, reaching a final concentration of 7.5 μM in a 1.5-ml Eppendorf tube. The sortase-mediated labeling reaction proceeded overnight at 4°C. Removal of excess sortase and unreacted protein was achieved using Ni-NTA beads. To eliminate excess DFO, a PD-10 column was used. Last, 20 kDa PEG DBCO was introduced to the protein solution for installation on the probe through a click reaction. The success of PEGylation onto the protein was confirmed via SDS-PAGE analysis.

### CAR T cell production

Murine CAR T cells were produced as described previously (*66*). Briefly, CD8^+^ T cells were isolated from the spleens of 14-week-old male or female C57BL/6 mice (the Jackson Laboratory) using Miltenyi Biotec CD8a (Ly-2) MicroBeads for mouse (positive selection kit; Miltenyi, 130-117-044) and LS columns (Miltenyi, catalog no. 130-042-401) as per the manufacturer’s instructions. The isolated T cells were cultured at 1 × 10^6^ cells/ml on six-well plates coated with anti-murine CD3e and anti-murine CD28 activating antibodies (Bio X-Cell, BE0001-1 and BE0015-1) in T cell media. After 24 hours, activated T cells were collected, counted, and resuspended at 0.5 × 10^6^ in a 50:50 mixture of fresh T cell media with viral supernatant supplemented with protamine sulfate to a final concentration of 10 μg/ml (MS Biomedicals, ICN19472910). A GFP was incorporated into the CAR construct using a P2A cleavage site (CAR-P2A-GFP) within the MP71 retroviral backbone, which was used for CAR T cell generation. The cells were spin-infected at 1000*g* for 1.5 hours at 37°C on new antibody-coated plates. The next day, T cells were again collected, counted, and resuspended at 1 × 10^6^ cells/ml in fresh T cell media, replated on new antibody-coated plates. Last, 24 hours later, the T cells were collected, counted, and the percentage of CAR^+^ (GFP^+^) T cells determined by flow cytometry. The desired number of CAR T cells was then prepared for injection by resuspension in saline and injected via tail vein. Alternatively, T cells were resuspended at this step in T cell media and plated for in vitro killing assays. The transduction rates of CAR T cells vary between batches, usually averaging around 50 to 60%. Examples of transduction rates for both CD19 CAR and control CAR are illustrated in fig. S10.

Human CAR T cells were made following a similar protocol. In brief, human PBMCs were isolated from apheresis leukoreduction collars. These cells were then mixed with anti-human CD3 and anti-human CD28 antibodies (BioLegend, no. 317326 and no. 302902) in addition to IL-2 (TECIN Teceleukin, Ro no. 23-6019), IL-15, and IL-7 (NSC nos. 745101 and 780247) for 24 hours. The next day, the cells were then mixed with viral supernatant, fresh IL-2, IL-15, and IL-7, and spin-infected at 2000*g* for 2 hours at 30°C. This step was then repeated on the following day, with fresh viral supernatant and cytokines. Last, 24 hours later, the cells were resuspended in fresh RPMI 1640 media and cytokines, and the percentage of CAR^+^ was determined through flow cytometry.

### In vitro killing assays

Briefly, target cells were counted and cocultured with or without CAR T cells at specified E:T ratios, accounting for the CAR T transduction efficiency, in T cell media. After approximately 24 hours, the cell suspension was subjected to flow cytometry analysis to evaluate live/dead status [via 4′,6-diamidino-2-phenylindole (DAPI) stain], %hCD19^+^ cells (anti-human CD19 BV785; BioLegend no. 302239), and %CD8^+^ cells (anti-mouse CD8-PE/Cy7; BioLegend no. 100722). Flow cytometry was also used to determine the densities of each cell type (CAR T, target cell, and non-transduced T cell). The resulting target cell densities in CAR T–containing wells were normalized to those in control wells, seeded with the same number of target cells but with control CAR T or non-transduced T cells. All flow cytometry experiments were conducted with a minimum of 10,000 live cells (via DAPI exclusion) and subsequent data analysis.

### IFN-γ and TNF-α release ELISA assays

The enzyme-linked immunosorbent assay (ELISA) followed standard procedures. Briefly, supernatants from in vitro CAR T cytotoxicity assays were collected and centrifuged to eliminate any contaminating cells. The quantification of TNF-α released by CAR T cells in the supernatant was performed using the mouse TNF-α ELISA kit (R&D systems, SMTA00B) according to the kit’s instructions. The quantification of IFN-γ released by CAR T cells in the supernatant was performed using the DuoSet ELISA kit for mouse IFN-γ (R&D systems, DY485). Nunc MaxiSorp flat-bottom plates (Thermo Fisher Scientific, 44-2404-21) were used for the assay, conducted on a Tecan Infinite 200 Pro machine according to the manufacturer’s instructions. To maintain the assay within the linear range of the kit, the supernatant was initially diluted at 1:10 in reagent diluent. Subsequently, a minimum of six serial fourfold dilutions were executed. For each plate, at least one standard curve was generated, and the entire experiment included at least two standard curves, constructed using standard solutions supplied by the manufacturer. The substrate solution used was 1-StepTM Ultra TMB-ELISA (Thermo Fisher Scientific, 34028), and the stop solution used was 2 N sulfuric acid (VWR, BDH7500-1). Bovine serum albumin (Sigma-Aldrich, A8022-500G) was prepared as a sterile-filtered 5% stock in phosphate-buffered saline (PBS, Corning, 21-031-CV).

### Mouse maintenance and studies

All animal studies were conducted in compliance with approved protocols from the MIT Committee on Animal Care (protocol number 0521-028-24). The mouse strains used in this study included C57BL/6 (the Jackson Laboratory). Immunocompetent C57BL/6 mice underwent sublethal irradiation (1 × 5 Gy) immediately before the transplantation of B-ALL cells. The B-ALL cells for transplantation were suspended in saline and administered via tail vein injection using 29-gauge syringes. Subsequently, mice received CAR T cells via tail vein injection, using 29-gauge syringes, 2 or 3 days later, as indicated in the results.

### Probe binding experiments in vitro

To assess the binding efficiency of the CD19 probe to CAR T cells in vitro, 1 × 10^5^ CAR T cells were subjected to incubation with varying concentrations (ranging from 0 to 100 nM) of the CD19 probe, which was either conjugated to Alexa647, DFO, or DFO along with nonradioactive Zirconium (Zr). The binding of hCD19 probes coupled to DFO or DFO and cold Zr was determined using anti-FLAG tag antibody APC (BioLegend, no. 637308). Triplicate analyses were conducted for all probe concentrations.

### Probe stability experiments in serum

The nonradioactive Zr-labeled DFO-PEG-CD19 probe was added to freshly obtained murine serum at a concentration of 1 μM and stored at 37° and at 4°C. Samples were collected at 6, 24, 48, and 72 hours and flash-frozen until all time points were collected. On day 3, all samples were thawed and used to stain CD19 CAR expressing Jurkat T cells. Secondary staining was performed with anti-FLAG antibody AlexaFluor 647. A fresh sample of the probe served as a positive control.

### Probe experiments in vivo

In vivo probe binding experiments were conducted using C57BL/6 mice. The mice underwent sublethal irradiation (1 × 5 Gy) and were transplanted with 3 × 10^6^ B-ALL cells via tail vein. Subsequently, 10 × 10^6^ CAR T cells were injected via tail vein 3 days after transplantation. After 2 days, the mice received an injection of 5 μg of Alexa647-labeled CD19 probe via tail vein. Five hours after probe injection, the mice were euthanized, and blood, spleen, and bone marrow were collected for flow cytometric analysis. Flow cytometry was used to determine the percentage of CD19 CAR T cells (GFP^+^) bound to the CD19 probe (Alexa647).

In survival experiments, C57BL/6 mice underwent sublethal irradiation (1 × 5 Gy) and were transplanted with 3 × 10^6^ B-ALL cells via tail vein. Two days after transplantation, 10 × 10^6^ CAR T cells were injected via tail vein. Three days later, mice received an injection of 5 μg DFO-PEG20–labeled CD19 probe coupled to cold Zr. Cold Zr labeling was achieved by incubating the CD19 probe with ZrCl_4_ solution (molar ratio CD19 probe to ZrCl_4_ = 1:3) for 1 hour before usage. Mice were euthanized at humane end points as defined by the Committee for Animal Care guidelines.

### Installation of ^89^Zr isotope to the CD19 probe and PET imaging

For installation of the ^89^Zr isotope to the DFO-labeled CD19 probe, ^89^Zr in 1 M oxalic acid was obtained from Washington University in St. Louis (Mallinckrodt Institute of Radiology). A working stock was created by neutralizing the 1 M oxalic acid with a half volume of 2 M Na_2_CO_3_ followed by 0.5 M Hepes buffer. Subsequently, an appropriate amount of the working stock was measured and mixed with the DFO-labeled CD19 probe, incubated on a shaker at room temperature for about 2 hours. The ^89^Zr-CD19 mixture was then filtered through a PD-10 column (Cytiva 17-0851-01) to remove unbound isotope, and radiolabeled fractions were collected and pooled for injection. Radio TLC analyses was used to confirm the purity of the radiolabeled probe (fig. S6). For radio TLC analysis, 1 μl of 1 mCi/ml free ^89^Zr in 1× PBS was spotted on iTLC-SA paper (Agilent, A120B12) and developed with 0.1 M citrate buffer (pH 5) as the mobile phase, which traveled 80 mm from the origin (10 mm).

PET images were acquired using a Sofie G8 PET machine. Static scans were conducted with a 10-min PET acquisition time followed by a 2-min μCT. Images were reconstructed using the internal reconstruction method (MLEM 3D) and output into a DICOM stack.

### PET experiments

For PET imaging experiments, C57BL/6 mice were sublethally irradiated (1 × 5 Gy) and transplanted with 3 × 10^6^ B-ALL cells via tail vein. Three days later, 10 × 10^6^ CAR T cells were injected via tail vein. After an additional 2 days, the mice were injected with about 5 μg CD19 probe coupled with ~50 μCi ^89^Zr via tail vein. Blood samples were collected at different time intervals (5, 10, 15, and 30 min and 1.5, 3, 4, 6, 16, 24, and 48 hours after injection) to determine the probe’s circulation half-life using a gamma counter. The mice were imaged 24 hours after probe injection. To determine optimal image time points, the mice were imaged at 24, 48, and 96 hours after probe injection.

### PET image analyses

PET image analyses were conducted using VivoQuant software (2022, InVicro Solutions). PET signal intensity was measured as μCi/mm^3^. CT scans were overlaid with PET signal to guide the generation of three-dimensional regions of interest (ROIs), which represented a certain organ within the mouse. The SUV_mean_ was calculated for this ROI using the mouse’s weight and radioactivity at the time of imaging: SUV = concentration in ROI/(activity at imaging/mouse body weight). To account for background signal differences, as in the case with our imaging time point optimization experiment, the SUV_mean_ of organs of interest was normalized against that of muscle, which should have no CD19-specific uptake. These results are presented as a unitless ratio. Quantification of radioactivity was decay corrected.

### Statistical analysis

All statistical analyses were conducted using GraphPad Prism 10 (GraphPad Software Inc). The specific statistical tests performed for each analysis are outlined in the figure legends. Differences were considered statistically significant for *P* values ≤0.05.
