# Supplementary material for: Development of an antigen-based approach to noninvasively image CAR T cells in real time and as a predictive tool
Source: Sci Adv. 2024 Sep 18;10(38):eadn3816. doi: 10.1126/sciadv.adn3816 (PMC11409975; doi:10.1126/sciadv.adn3816)
Supplement: Supplementary file 1 — Figs. S1 to S10 Legends for movies S1 to S9 Supplementary Text [file sciadv.adn3816_sm.pdf]

Science Advances
AAAS

Supplementary Materials for

Development of an antigen-based approach to noninvasively image CAR T cells in real time and as a predictive tool

Julia Fröse *et al.*

Corresponding author: Michael Hemann, hemann@mit.edu;
Mohammad Rashidian, mohammad_rashidian@dfci.harvard.edu

Sci. Adv. **10**, eadn3816 (2024)
DOI: 10.1126/sciadv.adn3816

The PDF file includes:

Figs. S1 to S10
Legends for movies S1 to S9
Supplementary Text

Other Supplementary Material for this manuscript includes the following:

Movies S1 to S9

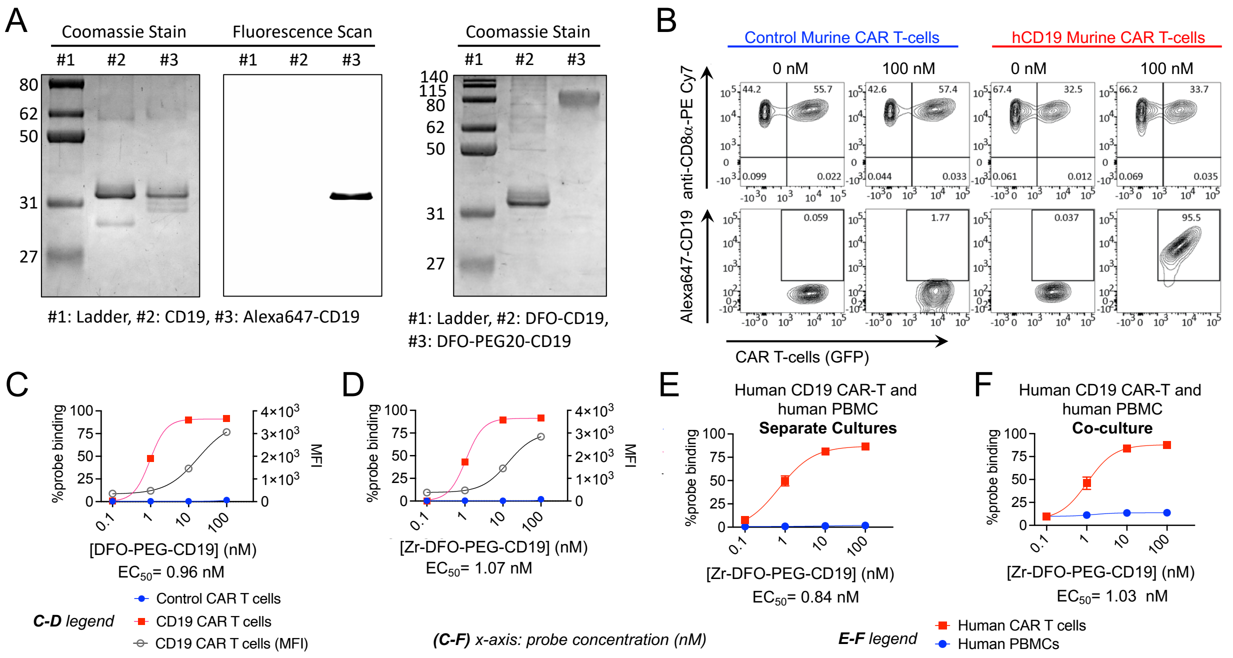

Fig. S1. Site-specific labeling and installation of DFO, PEG, and zirconium on the CD19 probe do not impact its binding and specificity. A) Characterization of site-specifically labeled CD19 with Alexa647, DFO, and PEG20 via SDS-PAGE analyses. Coomassie staining and Alexa647 fluorescence scans of the gels for Alexa647-CD19 (left and middle, respectively) and Coomassie staining of the gel for DFO-PEG20-CD19 (right) are presented. Lanes are labeled at the bottom of the gels. **B)** Analysis of the CD19 probe binding to murine CAR T-cells. 100,000 CAR T-cells were incubated with various concentrations of Alexa647 labeled CD19 probe and subsequently stained for CD8α as discussed in Fig. 1D in the manuscript. Flow cytometric analysis was conducted after a 30-minute incubation, gating on CD8α^{+} and GFP^{+} CAR T cells. The gating strategy for flow cytometric analysis of Alexa647-CD19 probe binding to control (EGFRvIII) and hCD19 murine CAR T cells is displayed for the 0 and 100 nM concentrations. The staining for varying doses of the probe is shown Fig. 1D in the main manuscript. **C-D)** Dose-dependent analysis of the DFO-PEG-CD19 (C) and Zr-DFO-PEG-CD19 (D) probe binding to hCD19 murine CAR T cells and control (EGFRvIII) murine CAR T cells (left y-axis) and dose-dependent Mean Fluorescent Intensity (MFI) (right y-axis). N=3 for each data point. The CAR T-cells are murine T cells transduced with a CAR construct containing an anti-human CD19 ectodomain or human EGFRvIII and a murine CD28-CD3z endodomain. **E-F)** Dose-dependent analysis of probe binding to human T-cell-derived CD19 CAR T-cells (CD19-41BB-CD3z CAR construct) and Hoechst-stained human PBMCs cultured separately (1:1 ratio, **E**) or together **(F)** with varying concentrations of the Zr-DFO-PEG CD19 probe. An anti-FLAG antibody was used for secondary staining of the DFO-PEG-CD19 probe. In the co-culture condition, staining of less than 10% of PBMCs is observed (F); however, this staining demonstrates no dose-dependence with the probe concentration. EC_{50} values are shown at the bottom of the graphs. All data were collected in triplicates, and error bars represent standard deviation.

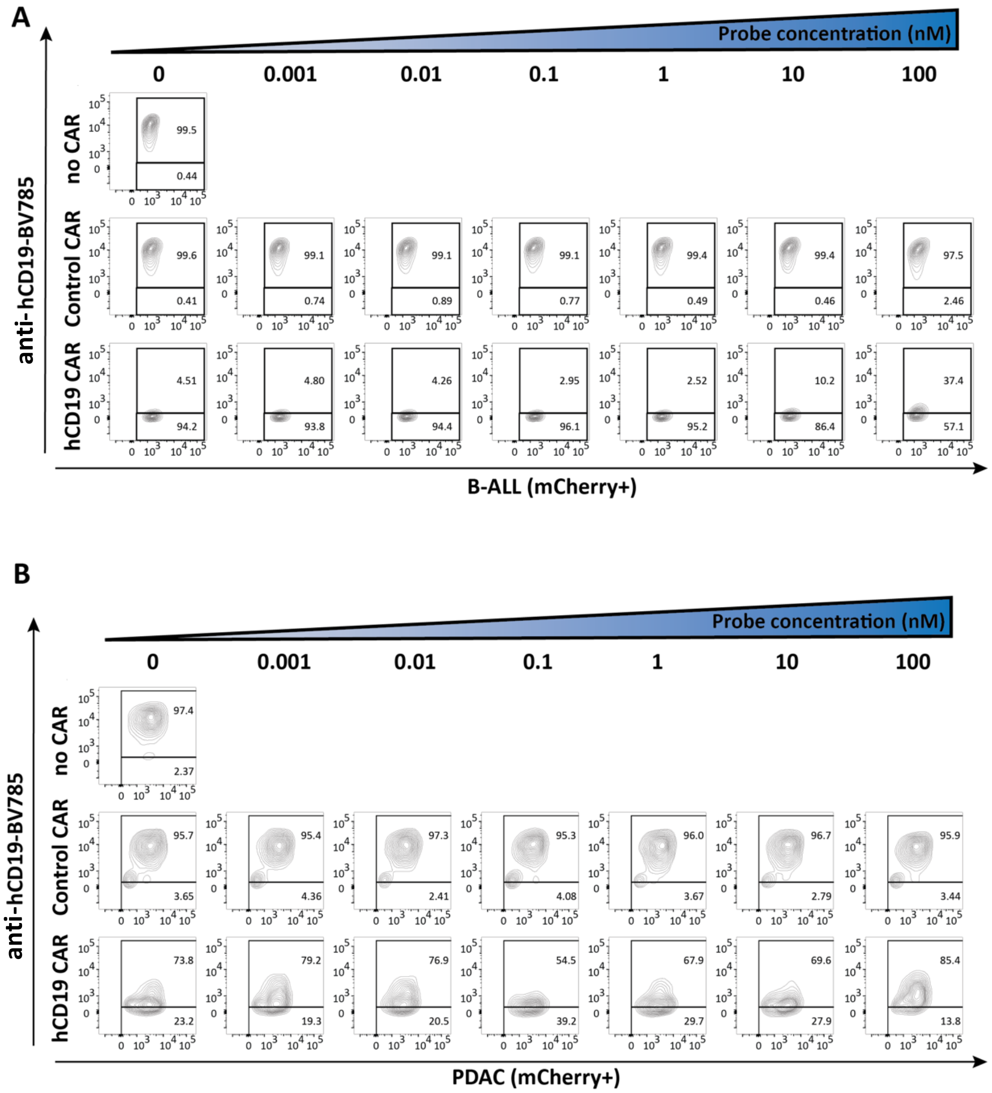

Fig. S2. Flow cytometric analysis of CD19 antigen expression on tumor cells co-cultured with anti-CD19 CAR T-cells in presence of the CD19 probe in vitro. A) Flow cytometric analysis of $hCD19^+$ B-ALL cells incubated for 24 hours with CAR T-cells (E:T ratio = 10:1) and increasing concentrations of the CD19 probe. The CD19 antigen was stained with anti-hCD19-BV785 antibody. Tumor cells are mCherry-positive. **B)** Flow cytometric analysis of $hCD19^+$ PDAC cells incubated for 24 hours with CAR T-cells (E:T ratio = 10:1) and increasing concentrations of the CD19 probe. mCherry-positive PDAC cells were stained for cell surface expression of hCD19 antigen with anti-hCD19-BV785 antibody. The CAR T-cells are murine T cells transduced with a CAR construct containing an anti-human CD19 or human EGFRvIII and a murine CD28-CD3z endodomain. Data are representative from one replicate, but all data was analyzed in triplicates.

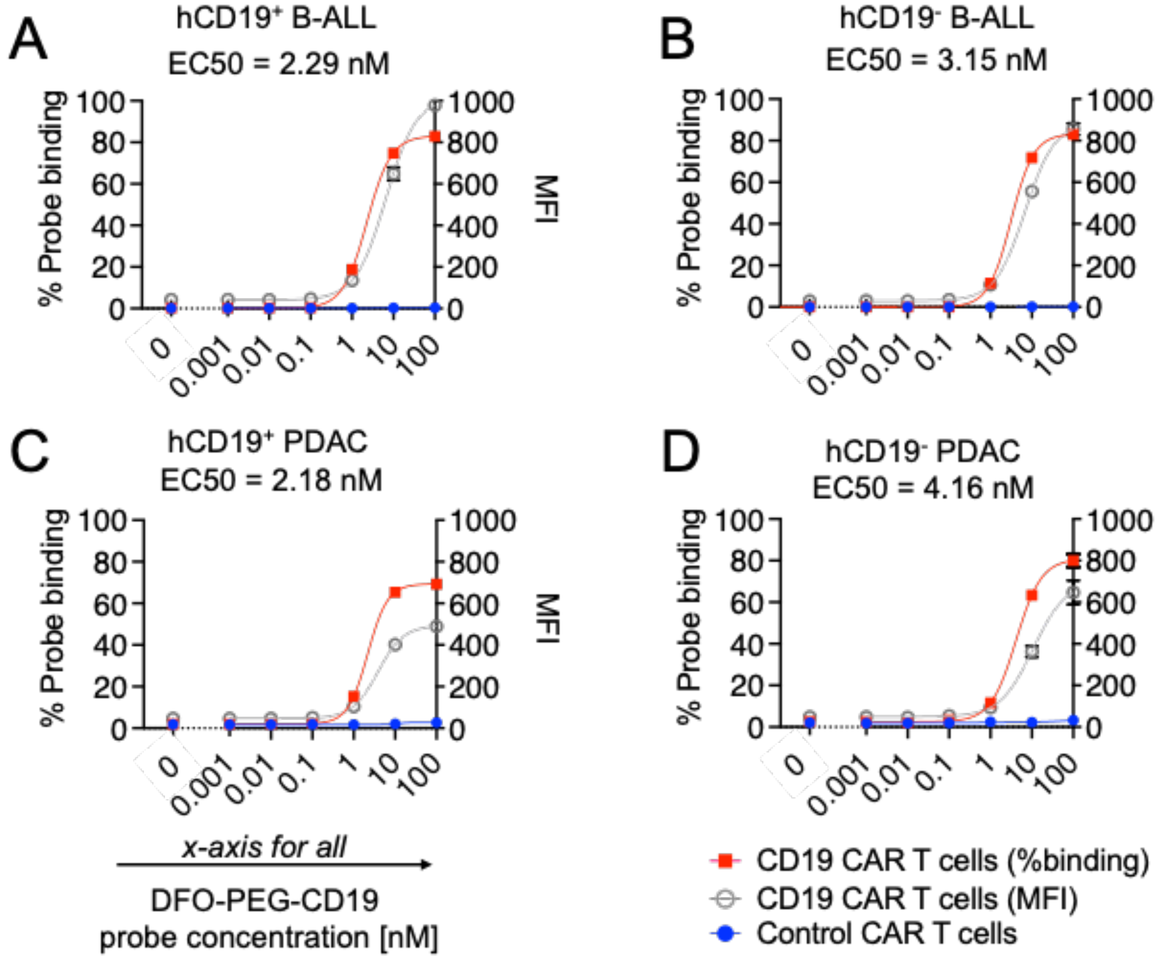

Fig. S3. The CD19 probe remains bound to CAR T-cells in the presence of tumor cells during an in vitro cytotoxicity assay, as shown in Fig. 3 in the manuscript. Shown are dose-dependent analysis of the DFO-PEG-CD19 probe binding to hCD19 CAR T-cells and control (EGFRvIII) CAR T-cells (left y-axis) and dose-dependent MFI of probe binding to hCD19 CAR T-cells (right y-axis). **A-B)** The CD19 probe was used during an *in vitro* assay with B-ALL cells (E-T ratio = 10:1) **(A)** expressing and **(B)** not expressing the human CD19 (hCD19) antigen. **C-D)** The CD19 probe was used during an *in vitro* killing assay with PDAC cells (E-T ratio = 10:1) **(C)** expressing and **(D)** not expressing the human CD19 (hCD19) antigen. A curve was fit for each binding curve and the EC_{50} was calculated using GraphPad Prism. All conditions were analyzed in triplicates. Error bars = SD. The CAR T-cells are murine T cells transduced with a CAR construct containing an anti-human CD19 ectodomain or human EGFRvIII and a murine CD28-CD3z endodomain.

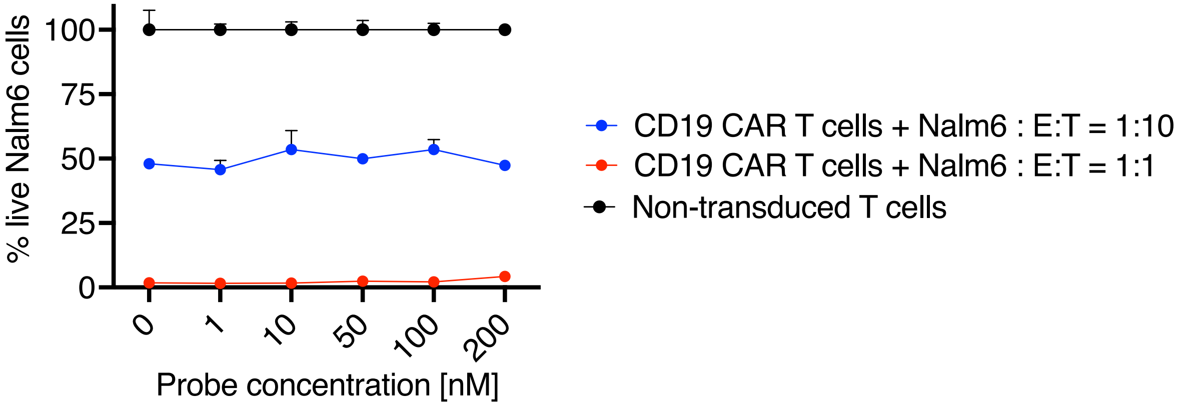

Fig. S4. The CD19 PET probe does not impair the cytotoxicity of human CD19 CAR-T cells in vitro. Human PBMC-derived CD19 CAR T-cells (CD19-41BB-CD3z CAR construct) were co-cultured with GFP^+ Nalm6 leukemia cells (Effector-to-Target ratio 1:1 and 1:10) alongside varying concentrations of the CD19 probe. Live Nalm6 cells were quantified after 24 hours by gating on GFP^+ cells. Non-transduced T cells served as an additional control. Error bars denote standard deviations.

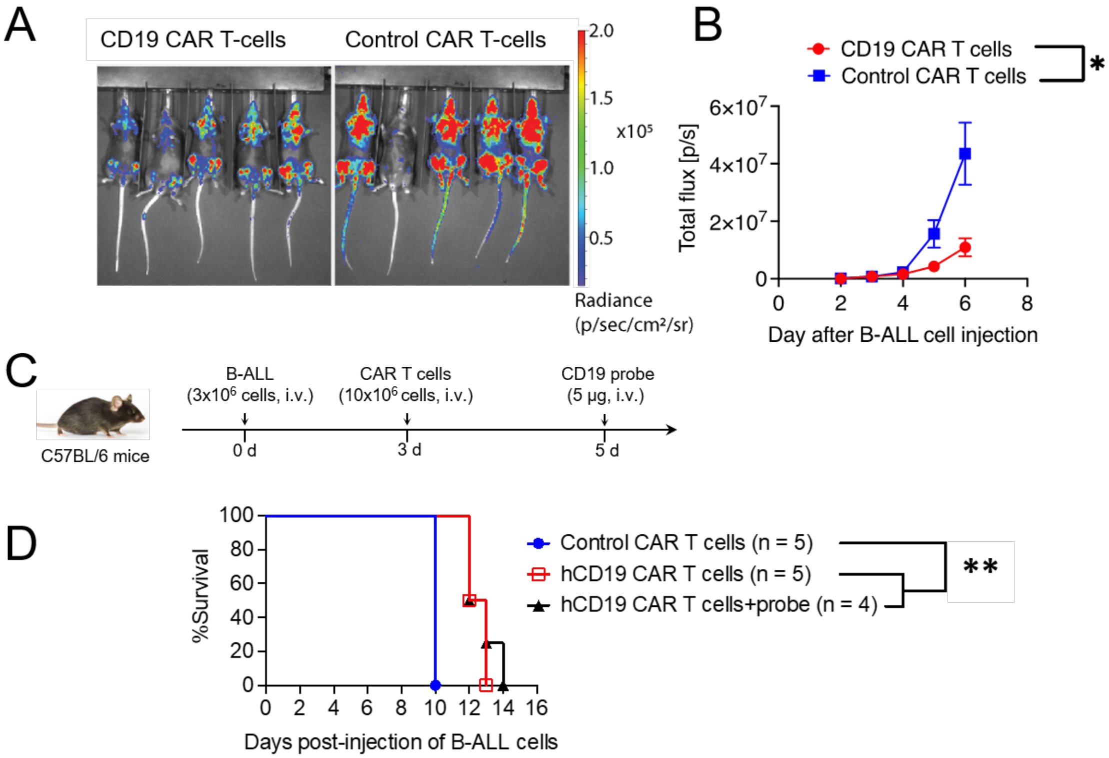

Fig. S5. The CD19 probe has no impact on CAR T-cell functionality in vivo. A-B) Daily bioluminescence imaging in mice with luciferase-expressing hCD19$^+$ B-ALL cells to monitor tumor burden during CAR T-cell treatment. Mice received injections of either 10x10^6 CD19 CAR T-cells or control CAR T-cells. **A)** Bioluminescent signal in mice six days after intravenous injection of 2x10^6 hCD19$^+$ B-ALL cells, and four days after receiving 10x10^6 hCD19 or control CAR T-cells. **B)** Quantitative analysis of bioluminescent imaging of B-ALL cells over time (n = 5 for each group). Error bars represent SEM. Student's t-test with p = 0.0202. **C)** Experimental timeline for hCD19$^+$ B-ALL, CAR T-cell, and probe injections in the survival experiment. **D)** Survival monitoring of mice injected with B-ALL and CAR T-cells alone or in combination with CAR T-cells and the Zr-DFO-PEG-CD19 probe until moribund state (n = 4-5). Mantel-Cox test with p = 0.0023. The CAR T-cells are murine T cells transduced with a CAR construct containing an anti-human CD19 ectodomain or human EGFRvIII and a murine CD28-CD3z endodomain.

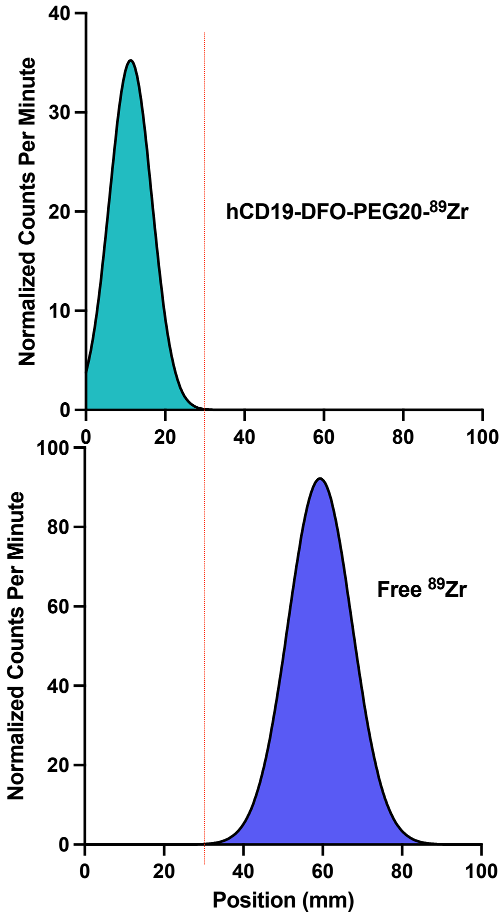

Fig. S6. Radio-TLC analysis of 89Zr-labeled CD19 probe. Radio-TLC analysis of hCD19-DFO-PEG20 probe following labeling with ^{89}Zr and purification with PD-10 column shows >99% purity. Count Per Minute (CPM) data for free ^{89}Zr and ^{89}Zr-DFO-PEG20-CD19 were normalized to the largest mean in each dataset (0-100%) and fitted with Gaussian nonlinear regression. The area under the curve (AUC) was calculated using GraphPad Prism (Version 10).

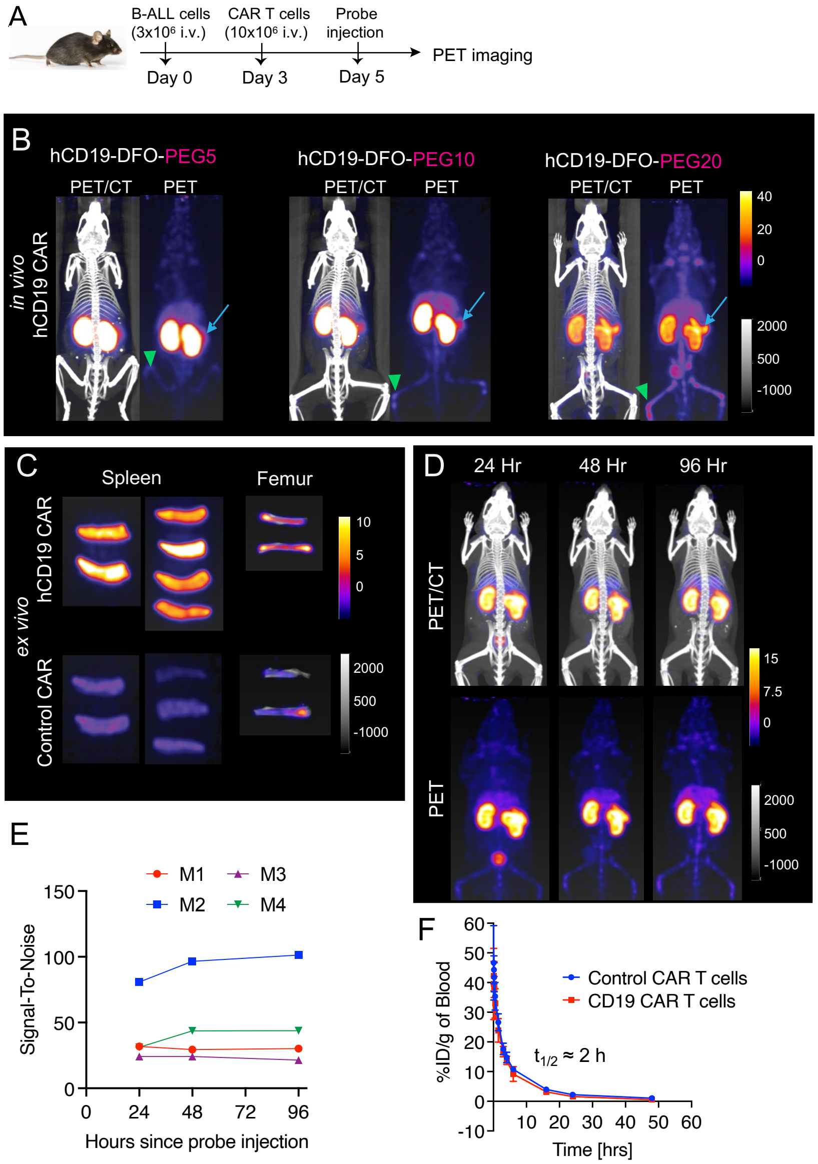

Fig. S7. Optimization and characterization of the ^{89}Zr-CD19 PET probe in vivo. A) Experimental outline for B-ALL, CAR T-cell and probe injections for PET imaging. **B)** Whole body PET/CT and PET images of CD19 CAR T-cells in B-ALL carrying mice using DFO-labeled CD19 probe with PEG5, PEG10 and PEG20 moieties at 24 hours after probe injection. All images have the same CT and PET settings for comparison. Spleen shown with blue arrow, femoral bone marrow shown with green arrowhead. **C)** *Ex vivo* PET images of spleens and bone marrow within the femur from B-ALL carrying mice with CD19 CAR T-cells and control CAR T-cells imaged with the CD19 PET probe 24 hours post-probe injection. **D)** Representative whole-body PET/CT (top panel) and PET (lower panel) images of CD19 CAR T-cells in B-ALL carrying mice at 24, 48 and 96 hours following ^{89}Zr-DFO-PEG20-labeled probe injection (n=4). **E)** Quantification signal-to-noise ratio of serial images of CD19 CAR T-treated mice at 24, 48 and 96 hours. Spleen SUV_{mean} served as signal measure, and gastrocnemius muscle SUV_{mean} served as noise measure for each mouse at each timepoint (n=4 total mice). **F)** Blood curve generated from serial sampling following ^{89}Zr-DFO-PEG20-labeled CD19 probe injection, measured with gamma counter (n = 4 - 5 for each group). The EC_{50} was calculated for probe clearance from mice injected with control CAR T-cells ($t_{1/2}$ = 103 min) or CD19 CAR T-cells ($t_{1/2}$ = 122 min).

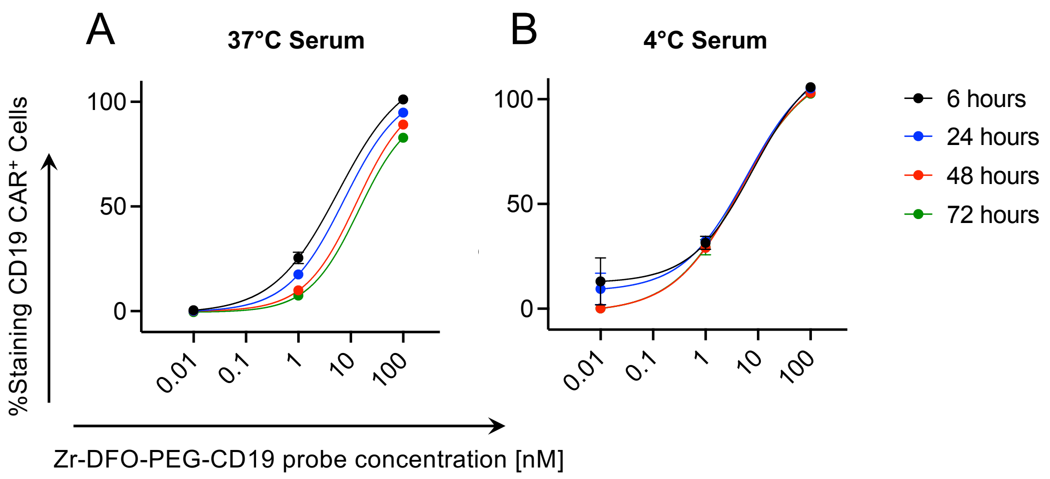

Fig. S8. CD19 probe is highly stable in serum at 37°C. The CD19 probe was added to freshly obtained murine serum at a concentration of 1 μM and stored at 37°C or 4°C. Samples were collected at 6 h, 24 h, 48 h, and 72 h and used to stain CD19 CAR^+ Jurkat T cells. A fresh sample of the probe served as a positive control which was used to normalize binding data. Staining of CD19 CAR^+ cells with probe incubated in serum at **(A)** 37°C and **(B)** 4°C demonstrated the probe is highly stable in serum through 72 h. The percentage of CD19 CAR T-cells stained over time showed a slight decrease in staining efficacy of the probe stored at 37°C in serum compared to samples stored at 4°C in serum. Error bars represent standard deviation for all graphs. N=3 for each condition.

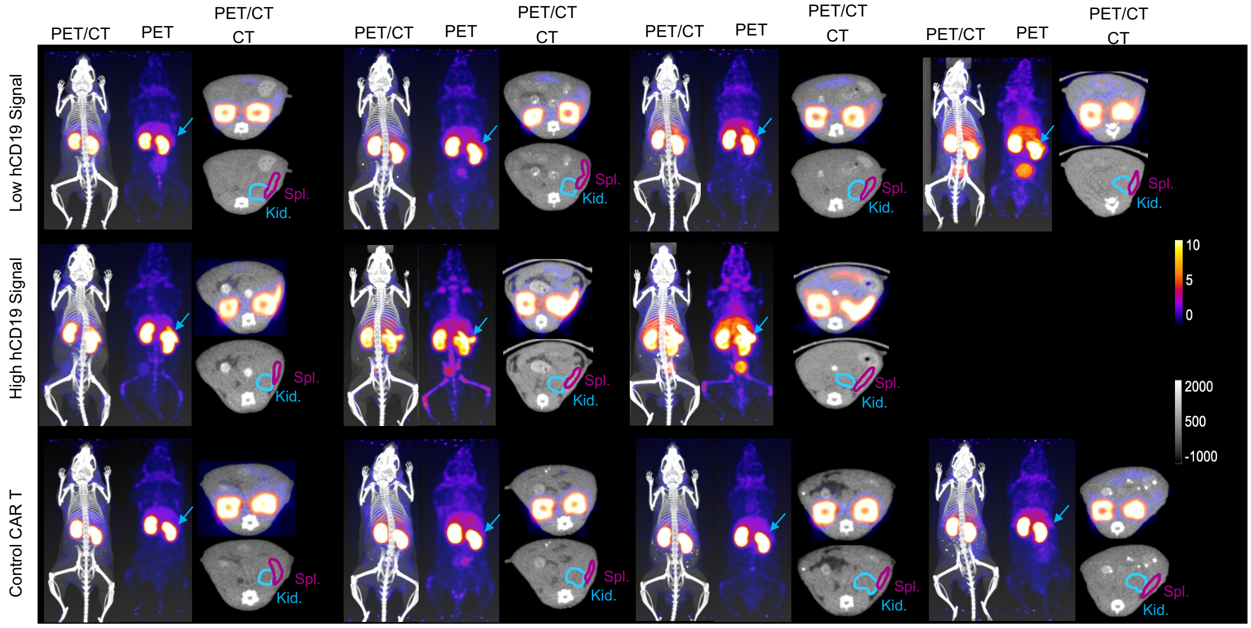

Fig. S9. The CD19 PET probe detects CD19 CAR T-cells in vivo and offers insights into the efficacy of CAR-T treatment in a syngeneic mouse model of B-ALL. PET-CT imaging of mice 24 hours after ^{89}Zr-DFO-PEG20-labeled CD19 probe injection, as shown in **Fig. 5** in the main manuscript. Left to right: Whole mouse CT scan overlaid with PET signal, PET image alone, and a transverse view of the mouse in CT only and PET/CT overlay at 24 hours post CD19 probe injection. Arrows indicate the spleen's location in the PET images, and circles denote the kidney and spleen in the transverse CT images.

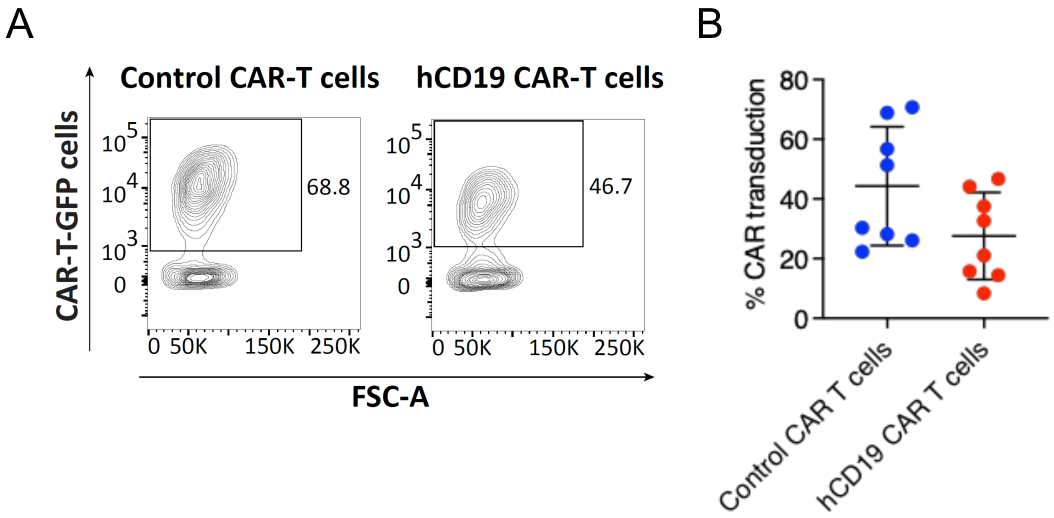

Fig. S10. CAR T-cell transduction rates. CAR T-cells were produced by transducing activated CD8 T cells with a hCD19 CAR or a control CAR (EGFRvIII)-P2A-GFP construct. The infection rates were determined using flow cytometry by gating on GFP$^+$ CAR-expressing cells. (**A**) Example of gating for GFP$^+$ CAR T-cells 48 hours after transduction of activated CD8 T cells with either hCD19 or control CAR-P2A-GFP construct. (**B**) Summary of CAR T-cell transduction rates for control and hCD19 CAR T-cells used in *in vitro* and *in vivo* experiments. Each data point represents the transduction rate for an individual CAR T-cell experiment. SD = standard deviation.

Movie Legends (S1-S9)

Movies S1-S3. The CD19 PET probe detects CD19 CAR T-cells in vivo in a syngeneic mouse model of B-ALL and can differentiate between different doses of CAR-T. C57BL/6 mice received an intravenous injection of 3 million murine B-ALL cancer cells expressing human CD19, followed by a subsequent intravenous injection of freshly prepared CD19 CAR T-cells in dose of 5 million (**S1**) or 10 million (**S2**) or control CAR T-cells (**S3**, dose of 10 million CAR). Mice were imaged with ^{89}Zr-DFO-PEG20 CD19 probe (5 μg, ~50 μCi, intravenous) three days after receiving CAR T-cells.

Movies S4-S9. The CD19 CAR-PET signal intensity predicts early mortality risk. Human CD19^{+} B-ALL C57BL/6 mice were pooled from two experiments using the same experimental set up described in Figure 4. Imaged mice showed exhibited heterogeneous CD19 CAR-PET signal. Mice injected with CD19 CAR-T were divided into two groups based on spleen SUVs: high CD19-PET signal (SUVs above 5.5, **S4-S5**) and low CD19-PET signal (SUVs below 5.5, **S6-S7**). Control mice are also shown (**S8-S9)**. For each mouse, CT/PET reconstructions were created with and without ipsilateral renal PET signal subtracted to allow improved spleen visualization.

Plasmids' constructs:

Plasmid	Producer
pCL-Eco (retroviral packaging)	Addgene plasmid #12371
psPAX2 (lentiviral packaging)	Addgene plasmid #12260
pMD2.G (VSV-g envelope)	Addgene plasmid #12259
hCD19 CAR-P2A-GFP plasmid	Reference (*66*)
EGFRvIII CAR-P2A-GFP plasmid	Reference (*66*)
pMMLV hCD19(-ICD)	Cloned as described below from VectorBuilder plasmid #VB181207-1130jpc

hCD19(-ICD) plasmid:

The pMMLV hCD19(-ICD) plasmid was produced by deleting most of the ectodomain of hCD19 by PCR from the pMMLV hCD19 plasmid (VectorBuilder). PCR was performed using Q5 polymerase (NEB M0491S) and the following primers:

hCD19(-ICD) Fwd: 5'-CCACCACCCAGCTTTCTTGTACAAAGTGGT-3'
hCD19(-ICD) Rev: 5'-TCAGGCGGCCGCGACCAGGGCTCTTTGAAGATGAAGA-3'

The finished product was PCR purified using the NEB Monarch PCR clean-up kit (NEB T1030S) and ligated using the DNA ligation kit, Mighty Mix (Takara 6023). The validity of the final product was confirmed using Sanger sequencing.

Sequence of the CD19 ectodomain used for imaging, as reported in reference (*62*). This sequence incorporates a few mutations compared to the wild-type CD19 protein, enhancing its stability. A FLAG tag (DYKDDDDK), a sortase recognition motif (LPETG), and a His6 tag were incorporated at the C-terminus of the protein.

GSPEEPLVVKVEEGDEAWLPCLKGTSDGPTQQLTWSRESPLKPFLKVSFGVPGLGVHVR
PNAVSLVISNVSQQMGGFYLCQPGPPSEKAWQPGWTVNVEGSGELFRWNVSDLGGLG
CGLKNRSSEGPSSPSGKLMSPKLYVWAKDRPEIWEGEPPCLPPRDSLNQSLSRDMTVAP
GSTLWLSCGVPPDSVSRGPLSWTHVHPKGPKSLLSLELKDDRPARDMWVTGTRLFLPR
ATAQDAGKYYCHRGNLTMSFHLEVKARPVSAHTKLRTGGWKGSDYKDDDDKLPETG
HHHHHH
